# Comparative phylogenetic analysis and transcriptional profiling of MADS-box gene family identified *DAM* and *FLC*-like genes in apple (*Malus*x *domestica*)

**DOI:** 10.1038/srep20695

**Published:** 2016-02-09

**Authors:** Gulshan Kumar, Preeti Arya, Khushboo Gupta, Vinay Randhawa, Vishal Acharya, Anil Kumar Singh

**Affiliations:** 1 Department of Biotechnology, CSIR-Institute of Himalayan Bioresource Technology, Palampur-176 061 (HP), India; 2 Academy of Scientific and Innovative Research, New Delhi, India

## Abstract

The MADS-box transcription factors play essential roles in various processes of plant growth and development. In the present study, phylogenetic analysis of 142 apple MADS-box proteins with that of other dicotyledonous species identified six putative Dormancy-Associated MADS-box (DAM) and four putative Flowering Locus C-like (FLC-like) proteins. In order to study the expression of apple MADS-box genes, RNA-seq analysis of 3 apical and 5 spur bud stages during dormancy, 6 flower stages and 7 fruit development stages was performed. The dramatic reduction in expression of two *MdDAMs*, *MdMADS063* and *MdMADS125* and two *MdFLC*-like genes, *MdMADS135* and *MdMADS136* during dormancy release suggests their role as flowering-repressors in apple. Apple orthologs of Arabidopsis genes, *FLOWERING LOCUS T*, *FRIGIDA*, *SUPPRESSOR OF OVEREXPRESSION OF CONSTANS 1* and *LEAFY* exhibit similar expression patterns as reported in Arabidopsis, suggesting functional conservation in floral signal integration and meristem determination pathways. Gene ontology enrichment analysis of predicted targets of DAM revealed their involvement in regulation of reproductive processes and meristematic activities, indicating functional conservation of SVP orthologs (DAM) in apple. This study provides valuable insights into the functions of MADS-box proteins during apple phenology, which may help in devising strategies to improve important traits in apple.

In plants, MADS-box transcription factors (TFs) play variety of roles in various biological processes during growth and development. MADS-box gene family is one of the best studied gene families in plants and contributes majority of component to the well-known ABCDE model of flowering that depicts their roles in floral organ development. However, MADS-box genes were also found to be expressed in vegetative tissues, ovule, embryo, root, trichome and fruit, suggesting their diverse roles in plant development^1^.The MADS-box proteins contain a highly conserved 58 amino acid long DNA binding MADS domain. The term MADS domain was derived from four members of the family *viz. MINICHROMOSOME MAINTENANCE1* (*MCM1*) from *Saccharomyces cerevisiae*, *AGAMOUS (AG*) from Arabidopsis, *DEFICIENS* (*DEF*) from *Antirrhinum majus* and *SERUM RESPONSE FACTOR* (*SRF*) from *Homo sapiens*^1^. Most of the studies on functional characterization of MADS-box genes have been done in model plant, *Arabidopsis thaliana*. However, studies on other flowering plant species like, *Antirrhinum majus*^2^, *Solanum lycopersicum*^3^ and *Petunia hybrida*^4^ also contributed significantly to reveal the diverse functions of MADS-box TFs. Although, MADS box TFs were originally found to be involved in determining floral organ identity, several recent studies showed their various important roles throughout the plant life cycle^1^.

In plants of *Rosaceae* family, particularly in apple (*Malus* x *domestica*), winter dormancy affects the flowering time by regulating dormancy release. The winter dormancy breaks only after the acquisition of sufficient chilling hours, which is genotype dependent^5^,^6^,^7^. In flowering plants, transition from vegetative phase to reproductive phase is a critical developmental process, which involves numerous molecular events. Role of many transcription factors (TFs) including those belonging to the MADS-box TF family has been shown in transition from vegetative to reproductive phase. One of these MADS-box TFs controlling flowering time is *SHORT VEGETATIVE PHASE* (*SVP*). The *SVP* mutant of *Arabidopsis* was shown to flower very early as compared to wild type, suggesting its role as a repressor of flowering^8^. The *SVP* was shown to repress flowering by regulating the expression of three flowering promoting genes namely; *FLOWERING LOCUS T* (*FT* ), *TWIN SISTER OF FT* (*TSF* ), and *SUPPRESSOR OF OVEREXPRESSION OF CONSTANS 1* (*SOC1*). In *Arabidopsis*, the time of flowering in response to ambient temperature is also governed by *SVP* through repression of *FT* expression^9^,^10^. Another MADS-box gene, *FLOWERING LOCUS C* (*FLC*) was also shown to act as a key flowering repressor, which interacts with *SVP* and inhibit the flowering by repressing the expression of *FT* and *SOC1* in different tissues^1^,^11^. The expression of *SVP* and *FLC* decreases gradually as plant becomes ready to flower^12^.

The dormancy insensitive ever growing peach mutant, which has mutation in *EVG* (*EVERGREEN*) locus, is the breakthrough in the dormancy studies in plants belonging to *Rosaceae* family, where deletion of tandem repeats of six orthologs of *SVP* was responsible for evergreen phenotype^13^. Therefore, these MADS-box genes were named as *Dormancy Associated MADS-box* (*DAM*) genes. The presence of homologs of *DAM* genes has also been reported in other *Rosaceae* plants like, raspberry, Japanese apricot and Japanese pears^14^,^15^,^16^. The expression pattern of *DAM* genes in various plants indicated role of low temperature in regulating their expression during dormancy period^7^,^17^,^18^. The ectopic expression of Japanese apricot *DAM6* in poplar was able to induce growth cessation of bud in transgenic popular^18^. These studies clearly showed that *DAM* genes play an important role in vegetative to reproductive transition.

Despite the vast functions of MADS box TFs in flowering plants, only few apple MADS-box TFs have been characterized, so far^19^,^20^,^21^,^22^. However recently, identification and expression analysis of apple MADS-box gene family in root, stem, leaf, flower and five stages of fruit development was performed^23^. Whereas in present study, we show that comparative phylogenetic analysis of apple MADS-box family proteins with that of other dicotyledonous species identified six putative *DAM* genes and four putative *FLC*-like genes in apple. Moreover, expression profiling of apple MADS-box family was performed during the key phenological events, including various stages of dormancy, dormancy release, flower and fruit development using RNA-seq approach. In addition, we have also identified putative downstream targets for DAM TFs in apple genome using computational approaches. Our analysis provides a basic framework to uncover the biological role of MADS-box TFs in various developmental processes during cyclic phenological events in apple.

## Results and Discussion

### Annotation of MADS-box family in apple

**Figure 1:**
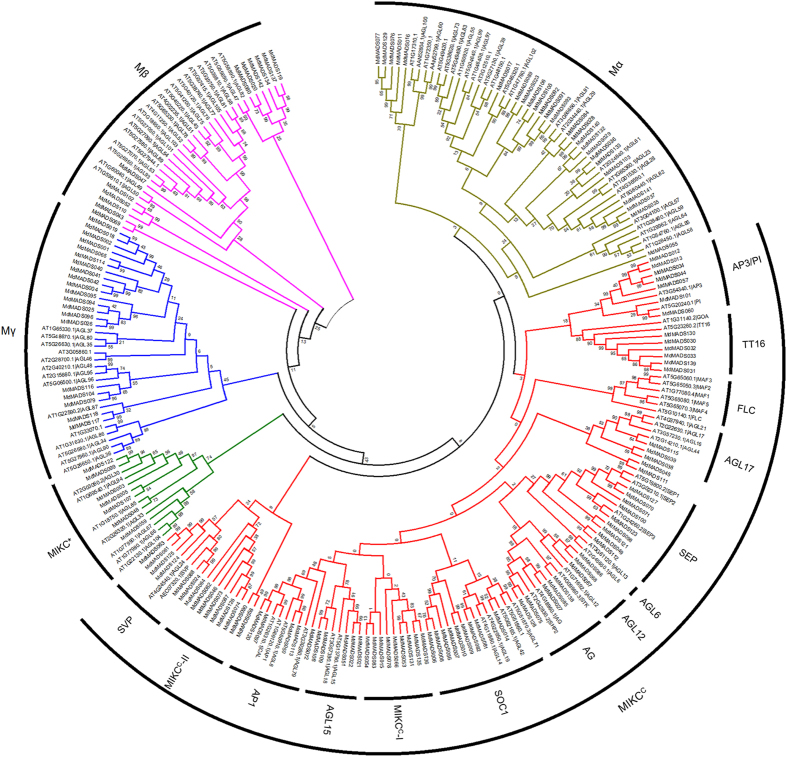
The unrooted phylogenetic tree of MADS-box proteins of apple and Arabidopsis. The multiple sequence alignment and construction of phylogenetic tree were performed with MEGA6.06 using neighbor-joining method with 1000 bootstrap replicates. The proteins were clustered and divided into five distinct sub-families. The MIKC^C^ sub-family was further divided into 14 sub-groups. The two sub-groups MIKC^C^-I and MIKC^C^-II seem to be apple specific.

The “hmmsearch” using HMM profile of MADS-box domain and PfamScan, with stringent e-value 1e-04, resulted in identification of 142 putative MADS-box proteins in apple (Supplementary Table S1). The number of proteins in apple MADS-box family was found to be second highest after soybean with 163 members^24^. However, a recent study by Tian *et al.*^23^ reported 146 proteins in apple MADS-box family. This slight variation in the number of MADS-box proteins may be due to the difference in approach used to identify the MADS-box proteins. The comparison of MADS-box proteins identified in the present and previous studies is listed in Supplementary Table S2. Phylogenetic tree made using full-length MADS-box proteins of Arabidopsis and apple showed the presence of previously defined sub-families, Mα, Mβ and Mγ for type-I and MIKC^C^ and MIKC* for type-II MdMADS proteins (Fig. 1). The three sub-families Mα, Mβ and Mγ contain 24, 12 and 20 proteins, respectively. Out of 86 type-II MdMADS, 79 were classified as MIKC^C^ and 7 as MIKC*. In addition, two sub-groups named MIKC^C^-I and MIKC^C^-II each containing 9 MdMADS were found to form the apple-specific clade with no Arabidopsis ortholog. The distribution of *MdMADS* genes on 17 apple chromosomes was found to be non-random showing chromosomal bias with highest (13) *MdMADS* genes on chromosome 5 and least number (1) on chromosome 3 (Supplementary Fig. S1). Out of 142 *MdMADS*, 75 (approximately 53%) genes were found to be present in 29 clusters distributed on 15 chromosomes. Interestingly, 17 out of 29 gene clusters containing a total of 45 *MdMADS* genes were found to be solely comprised of MIKC^C^ genes. Duplication analysis of *MdMADS* genes revealed the presence of 26.8%, 23.2%, 18.3% and 30.3% of segmental, tandem, dispersed and proximal duplication, respectively. In total, 18 type-I and 38 type-II *MdMADS* genes were found to be tandem (Supplementary Fig. S2) or segmental (Supplementary Fig. S3) duplicated. Therefore, expansion of type-II *MdMADS* genes might be due to the recent whole genome duplication during evolution^25^.

### Phylogenetic relationship of MdMADS family with that of other plant species

**Figure 2:**
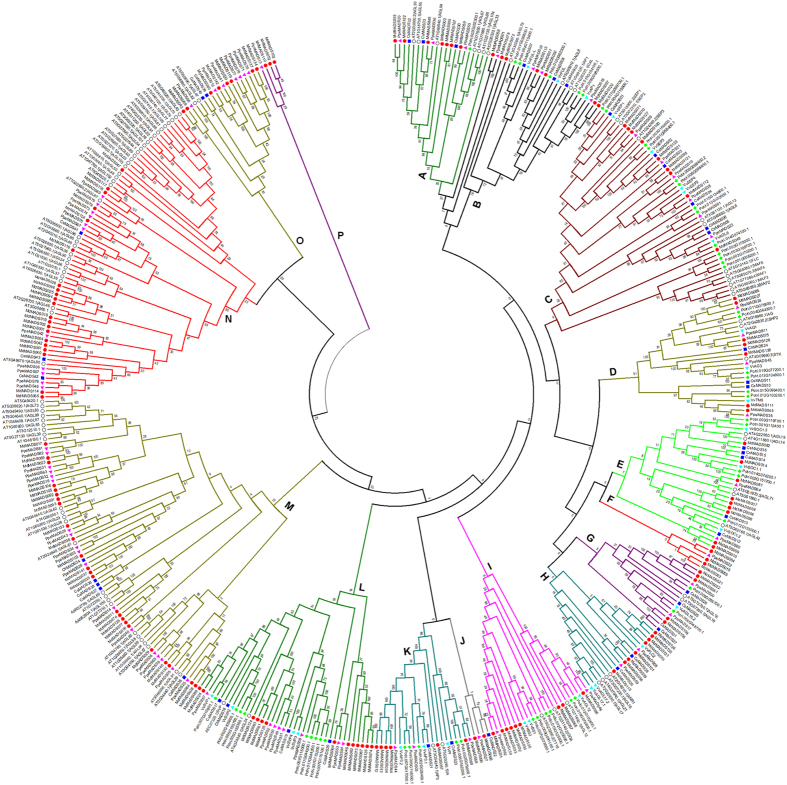
Phylogenetic tree of MADS-box proteins of apple with that of Arabidopsis, peach, poplar, cucumber and grape. The multiple sequence alignment and construction of phylogenetic tree were performed with MEGA6.06 using neighbor-joining method with 1000 bootstrap replicates. The tree was divided into 16 phylogenetic subgroups, designated as A to P.

To examine the phylogenetic relationship of MdMADS proteins with that of Arabidopsis, peach, poplar, cucumber and grape, phylogentic tree was made that divided all these MADS-box proteins in to 16 distinct clades (A to P; Fig. 2). Interestingly, in all the clades, MADS-box proteins of members of Rosaceae family (apple and peach) were clustered in separate sub-clades. The members of each MADS-box protein sub-family (Mα, Mβ, Mγ, MIKC^C^ and MIKC*) of different plant species were clustered in the same clade. For instance, members of Mα, Mγ and MIKC* sub-families were clustered in clade M, N and A, respectively. While, members of Mβ sub-family were clustered in clade O and P, and the remaining clades contain members of MIKC^C^ sub-family (Fig. 2).

Orthologs of SVP were reported to play important roles in dormancy establishment and its maintenance and were characterized as *Dormancy Associated MADS-box* (*DAM*) TFs in poplar^26^, peach^13^ and leafy spurge^17^. From the phylogenetic analysis, the expansion of SVP clade was observed in apple with six orthologs. In previous studies, the tandem duplication of *SVP* genes was found to be responsible for their expansion in apricot^27^ and peach^28^. However, none of the apple orthologs of *SVP* was found to be tandem duplicated, suggesting that tandem duplication did not play any role in their expansion. In apple, four genes (MDP0000322567, MDP0000259294, MDP0000209705 and MDP0000527190) were previously annotated as *DAM* in Genome database for Rosaceae (https://www.rosaceae.org). However, out of these four DAM genes, only three were found to exhibit dormancy associated expression, in transgenic apple over-expressing peach *CBF* gene, which were then annotated as *DAM1* (MDP0000322567), *DAM2* (MDP0000259294) and *DAM3* (MDP0000209705)^29^. However, the phylogenetic analysis of MdMADS with that of other dicotyledonous species resulted in identification of six putative DAM proteins in apple (Fig. 2). Although, only two MdMADS (MdMADS024; MDP0000209705 and MdMADS088: MDP0000233948) were found to be close orthologs of Arabidopsis SVP while, the remaining four (MdMADS061; MDP0000259294, MdMADS063; MDP0000527190, MdMADS124; MDP0000255146 and MdMADS125; MDP0000322567) were clustered in sub-clade along with the peach DAM proteins (Supplementary Fig. S4), therefore these six apple proteins were designated as MdDAMs. This observation suggests that DAMs are conserved in Rosaceae family during the course of evolution indicating the functional conservation of DAM proteins in peach and apple.

Similarly, the FLC was reported to be involved in vernalization in Arabidopsis to initiate flowering^1^. Phylogenetic analysis of MdMADS with their Arabidopsis counterparts did not identify any ortholog of Arabidopsis FLC and MAF in apple (Fig. 1). Similarly, no ortholog of *FLC* has been reported in apricot^27^ and cucumber^30^. However, *FLC*-like genes have been reported in other plants like poplar^26^, peach^28^ and grapevine^31^. Interestingly, the phylogenetic analysis of MdMADS with that of other dicotyledonous species indicates that four MdMADS (MdMADS053, MdMADS131, MdMADS135 and MdMADS136) were clustered in the clade H along with FLC-like proteins of peach and grape (PpeMADS08, VvFLC2); that suggests the presence of putative FLC-like MdMADS in apple (Fig. 2). Therefore, these four proteins were designated as FLC-like MdMADS. A recent transcriptome study in apple reported the presence of putative *FLC*-like gene (MDP0000207984), however it was not functionally characterized^32^. In the present study, that gene was not identified as *MdMADS* due to the absence of MADS domain.

### Analysis of expression pattern of *MdMADS* genes in various developmental stages during apple phenological cycle

To examine the expression of *MdMADS* genes in various developmental stages of different tissue types, RNA-seq analysis of 21 samples representing 3 stages of apical bud, 5 stages of spur bud, 6 stages of flower development and 7 stages of fruit development (Supplementary Table S3) was performed. The paired-end sequencing generated 264,658,274 raw reads of which 17,227,734 low quality reads were filtered out. The *de novo* assembly of 247,430,540 high quality reads resulted in 64,669 contigs. Out of 142 *MdMADS*, 63 were found to be expressed in various tissues, while rest of the 79 genes exhibit either very low or no expression in any of the tissues.

### Type-I MADS genes

**Figure 3:**
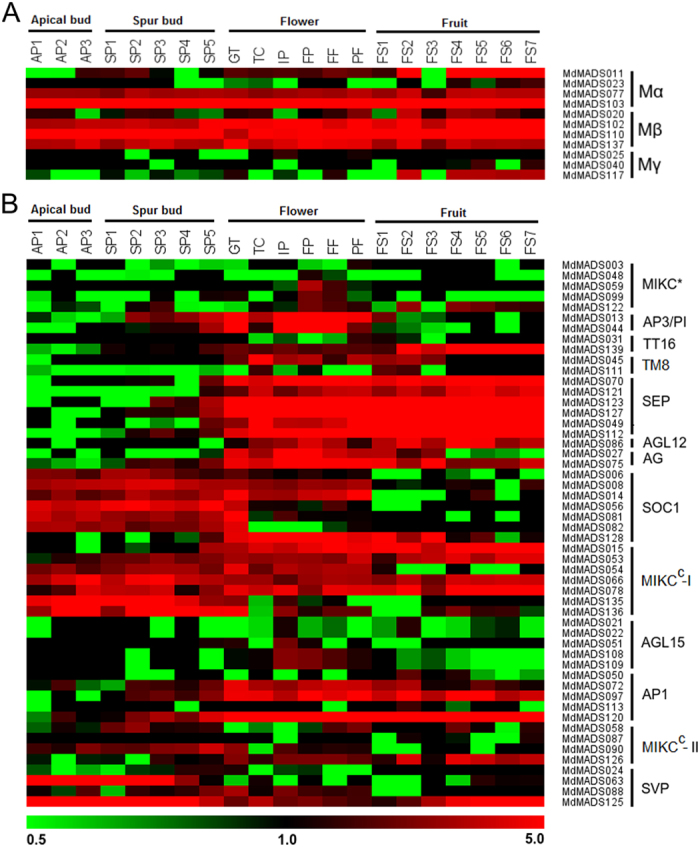
The heat map representation of *MdMADS* genes. (**A**) type-I and (**B**) type-II *MdMADS-box* genes in various developmental stages of bud, flower and fruit tissues. The log2 transformed FPKM values were used to generate the heat-map using MeV4.

In case of type-I *MdMADS* genes, only 11 out of 57 were found to be expressed in RNA seq data. The 3 genes *MdMADS102*, *MdMADS110* and *MdMADS137* of Mβ sub-family and two genes *MdMADS077* and *MdMADS103* of Mα sub-family were found to be ubiquitously expressed in all the samples analyzed (Fig. 3A). Recent studies showed involvement of type-I *MADS-box* genes in plant reproduction processes^25^,^33^,^34^. A member of Mγ sub-family in Arabidopsis, AGL80, which interacts with AGL61 of Mα sub-family was found to be involved in female gametophyte development^1^. In the present study, ortholog of *AGL61*, *MdMADS103* was found to exhibit expression in all the tissues, while no close ortholog of *AGL80* was found to be expressed. In addition, the genes of Mα and Mγ showed variable expression during fruit development (seed development along with the fruit development). Similarly genes of these sub-families were also found to be expressed during seed development in Arabidopsis^35^.

### Type-II MADS genes

#### MIKC*

The expression of type-II MADS genes was found to be high in most of the tissue types, suggesting their role in diverse developmental processes in apple (Fig. 3B). The MIKC* genes in Arabidopsis have been found to control the pollen development^36^. Similarly in peach, the expression of *PpeMADS20*, *PpeMADS36* and *PpeMADS55* was found to be high in pollens during flower development^28^. The close orthologs of these genes in apple, *MdMADS099*, *MdMADS48* and *MdMADS59* also exhibit higher expression at later stages (FP and FF) of flower development (Fig. 3B). In contrary, Tian *et al.*^23^ did not find expression of these genes in flower tissue. This may be due to the difference in flower stage at which sample was collected.

#### Floral identity genes

Previous studies have shown the role of MIKC^C^ genes as flower homeotic genes in Arabidopsis and other plants leading to establishment of ABCDE model of floral organ identity^1^. In Arabidopsis, the genes of AP1 clade, *AP1* and *CAL* play role in petal and sepal identity with additional role of *FUL* in fruit development^1^. In the present study, close orthologs of *AP1* and *CAL* in apple, *MdMADS097* and *MdMADS120* were found to exhibit high expression during flower and fruit development. Similar expression pattern of these genes was also observed by Tian *et al.*^23^. Previously, Cevik *et al.*^22^ reported the involvement of *FUL*-like gene in apple fruit development. Similarly in peach, *FUL* ortholog, *PpeMADS37* was found to be expressed in fruit tissue^28^. Although, *MdMADS050* and *MdMADS113*, close orthologs of *FUL*, were found to be expressed during initial stages of fruit development (FS2) in present study (Fig. 3B), while no expression of *MdMADS050* was reported by Tian *et al.*^23^ in fruit tissue. Expression of genes of AP1 clade in flower as well as in fruit tissues might be due to fact that apple is not a true fruit and develops from the enlargement of basal part of sepal.

In Arabidopsis, the identity of petal and stamen is defined by *AP3* and *PI*^37^. Similarly, the high expression of genes of AP3 clade, *PpeMADS56* in peach^28^ and *PmMADS12*, *PmMADS13* and *PmMADS29* in apricot^27^ was also observed in flower tissue. In present study, two genes, *MdMADS013* and *MdMADS044* of AP3/PI clade were found to exhibit high expression during flower initiation and development (Fig. 3B), however, Tian *et al.*^23^ found expression of only *MdMADS013* in flower tissue. The similar spatial expression of AP3/PI clade genes in apple, peach and apricot indicates their functional conservation in higher woody plants. Moreover, the Arabidopsis genes of AG clade perform C function and D function of homeotic genes. In apple, *MdMADS027* a close ortholog of *AG* was found to be expressed in flower and initial stages of fruit development, while *MdMADS075* an ortholog of *SHP2* has expression in flower and fruit development stages (Fig. 3B), the similar expression patterns of these genes were observed by Tian *et al.*^23^ in flower and fruit tissues. Similar to apple, the members of AG clade were also observed to have high expression during fruit development in peach^28^ and apricot^27^. These finding suggest the possible role of AG class genes in development of pome and stone fruits.

#### Other MIKC^C^ genes

Role of *SEP* gene orthologs has been established in tomato^38^ (*TM29*) and strawberry^39^ (*FaMADS9*) fruit development. While in Arabidopsis, the *SEP* genes (E class genes) are involved in multimeric protein complex formation with other homeotic genes and specify the stamen, carpel and petal identity^40^. In peach, the expression of four *SEP* genes was found restricted to fruit tissues^28^. However in apricot, three orthologs of *SEP* have higher expression in fruit and flower tissues^27^. Similarly, in the present study, six *SEP* orthologs (*MdMADS049*, *MdMADS070*, *MdMADS112*, *MdMADS121*, *MdMADS123* and *MdMADS127*) have high expression in all the developmental stages from flower initiation to fruit development (Fig. 3B). A previous study in apple, also observed the expression of *SEP* ortholog during flower and fruit development^19^,^23^. In Arabidopsis, the functions of SEP genes were found to be largely similar to the functions of AP1 genes^41^. Interestingly, in the present study two orthologs of *AP1* (*MdMADS097* and *MdMADS120*) also have similar expression pattern to *SEP* orthologs (Fig. 3B), which indicates the conservation of functional redundancy of *SEP* orthologs in apple also.

The MIKC^C^ genes are also known to be involved in processes other than the flower organ identity. The Arabidopsis *AGL15* and *AGL18* genes are expressed in embryo and act as additional flowering repressor in a redundant manner^42^. In contrast, the *SOC1* gene regulates flowering time by preventing the premature differentiation of flower meristem and act as activator of flowering in vernalization and GA dependent pathway in Arabidopsis^43^. In apple, three members of AGL15 clade, *MdMADS051*, *MdMADS108* and *MdMADS109* were found to exhibit expression at later stages of flower development (time of embryo development), while in all other tissues their expression was negligible. The expression of seven members of SOC1 clade was high during the bud development stages, however *MdMADS08* and *MdMADS014* exhibit higher expression in flowering stages also (Fig. 3B), similarly, these genes were observed to be expressed in flower tissue by Tian *et al.*^23^. The *AGL12*, a sister gene of AG clade regulates root meristem growth in addition to promoting flowering in Arabidopsis^44^. In apple, *AGL12* ortholog, *MdMADS086* and *AG* ortholog *MdMADS075* exhibit similar expression in flower and fruit tissues (Fig. 3B). Therefore, the *AGL12* ortholog in apple might also have role in regulation of flowering. However, members of TM8 clade are not present in Arabidopsis^28^, similar to other dicotyledonous species, the interspecies phylogenetic analysis shows the presence of two members of TM8 clade in apple. The *MdMADS045*, a member of TM8 has high expression in flower development stages. While another TM8 member, *MdMADS111* showed low expression during flower development (Fig. 3B). The similar expression of these genes was also observed by Tian *et al.*^23^, however, these genes were grouped into SOC1 clade based on phylogenetic analysis with Arabidopsis which lack members of TM8 clade. The low expression of genes belonging to TM8 clade in flower tissue has also been reported in grape^31^ and peach^28^. In Arabidopsis, the *TT16* which is mainly expressed in ovule was shown to be involved in flavonoid biosynthesis in the seed coat^45^. In apple, we identified two members of TT16 clade (*MdMADS031* and *MdMADS139*), however only *MdMADS139* was expressed highly during fruit development.

#### FLC-like and DAM MADS-box TFs

**Figure 4:**
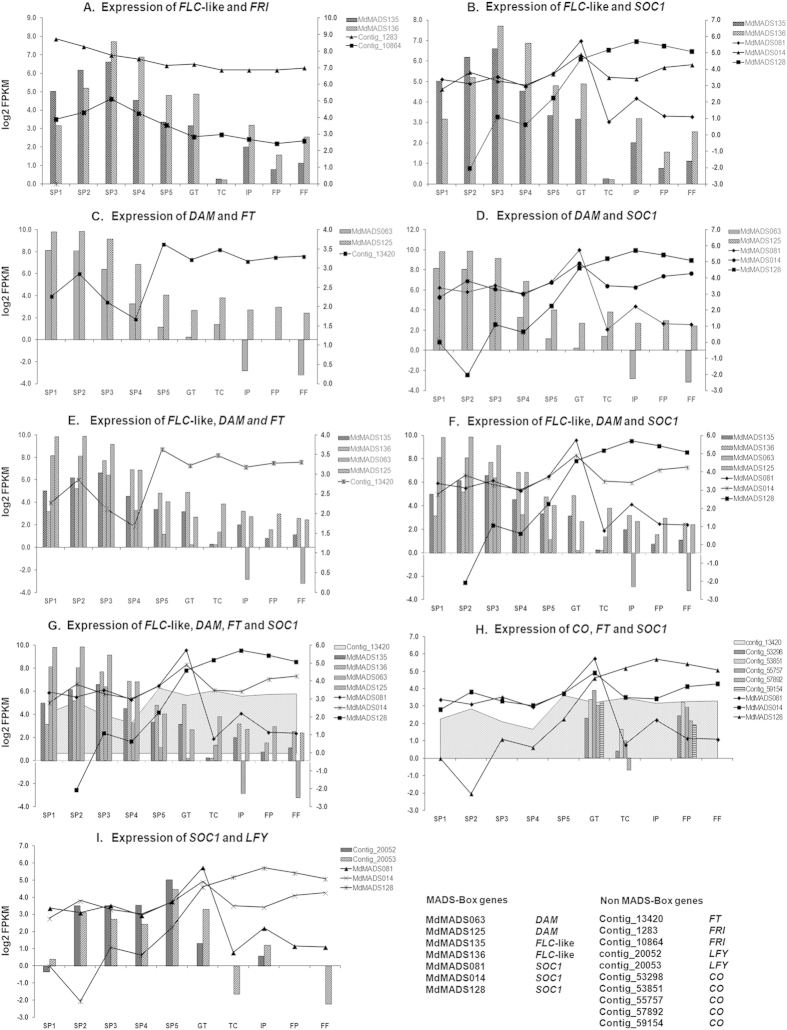
Comparative expression profile analysis of genes involved in floral signal integration. The expression profiles of *FLC*-like, *FRI*, *DAM*, *FT*, *SOC1*, *CO* and *LFY* genes in ten developmental stages of dormant bud to full bloom stage. The log2 transformed FPKM values were used to plot the graphs. The primary and secondary axis were used for column and area & line plots, respectively.

The role of *SVP* and *FLC* as flowering time regulators has been extensively studied in Arabidopsis and a model for flowering time regulatory network has been hypothesized that depicts the inhibitory effect of *FLC* and *SVP* on flowering through different pathways^1^,^43^. In order to elucidate the functional conservation of this hypothetical model in apple, the expression analysis of *SVP* orthologs (*DAM*), *FLC*-like genes and other genes associated with floral regulation was performed. In Arabidopsis, *FLC* act as an important flowering repressor and its expression is epigenetically downregulated after prolonged cold exposure during vernalization process^46^. Moreover interaction between FLC and SVP has been found to repress the expression of *FT* gene, a flower promoting gene^11^. In addition, the *FLC* has also been suggested to be involved in flower development and juvenile to adult phase transition^46^. In apple, the putative *FLC*-like genes *MdMADS135* and *MdMADS136*, identified through phylogenetic analysis (Fig. 2), exhibit increased expression with chilling acquisition during winter dormancy, while reduction in expression at the time of dormancy release (Figs 3B and 4A). Similar expression patterns of *FLC* in Arabidopsis^46^ and *FLC*-like gene in poplar^47^ have been reported. Therefore, we suggest that *MdMADS135* and *MdMADS136* may have similar role as negative regulator of flowering as defined in Arabidopsis and poplar. While another apple *FLC*-like gene, *MdMADS053* was expressed in most of the tissue types. The similar expression of *FLC*-like gene, *PpeMADS8* was also observed in peach^28^. In case of apple, *FLC*-like genes have shown high sequence divergence than that of Arabidopsis, which suggest their functional divergence. However, apple and poplar *FLC*-like genes exhibit dormancy associated expression. Moreover, *FLC* downregulates the expression of flower promoting genes *SOC1* and *FT* gene in Arabidopsis^1^,^11^. In present study, the reciprocal expression of *FLC*-like genes and orthologs of *SOC1* and *FT* was also observed during dormancy release and flower initiation (Fig. 4B,E). In addition, the expression of orthologs of *FRIGIDA* (*FRI*), an upstream positive regulator of *FLC*^43^, was found to be high during dormancy and reduced gradually with concomitant reduction in expression of *FLC*-like genes in apple (Fig. 4A). The similar expression patterns of Arabidopsis *FLC* and *FLC*-like genes of other dicotyledonous species indicate their functional conservation.

The *SVP* mutant of Arabidopsis flower earlier than the wild types, indicating its role in controlling vegetative to reproductive transition^8^. In peach, six tandemly duplicated orthologs of *SVP*, *DORMANCY ASSOCIATED MADS-BOX* (*DAM*) genes are involved in seasonal floral bud dormancy^13^. The phylogenetic analysis of MdMADS proteins with that of Arabidopsis and other dicotyledonous species also shows the presence of six SVP orthologs, DAM in apple (Fig. 2). The RNA-seq data showed that only four putative *MdDAM* genes (*MdMADS024, MdMADS063, MdMADS088* and *MdMADS125*) were found to be expressed of which *MdMADS063* and *MdMADS125* showed reduction in expression along with the dormancy release (Fig. 3C). Similar to *FLC*, the *SVP* also acts as negative regulator of *SOC1* and *FT* in Arabidopsis, therefore the expression of *SOC1* and *FT* orthologs in apple was analyzed in relation to *DAMs* and *FLC*-like genes (Fig. 3G). The expression of *MdDAM* and orthologs of *SOC1* and *FT* in apple was found to be reciprocal during dormancy to active phase transition (Fig. 4C,D). Similarly, reciprocal expression of *DAM* and *FT* genes was also reported in leafy spurge^17^. Repressive effect of *PmDAM6* on expression of *FT2* was also shown in transgenic poplar over-expressing *PmDAM6*^18^. In addition, expression of *DAM* was induced with dormancy inducing signals and concomitantly decreased with dormancy release in raspberry^14^, pear^16^, apricot^18^,^27^, peach^7^ and leafy spurge^17^. Therefore, expression pattern of *MdDAMs* indicates the functional conservation of *SVP* orthologs in apple and other dicotyledonous species.

**Figure 5:**
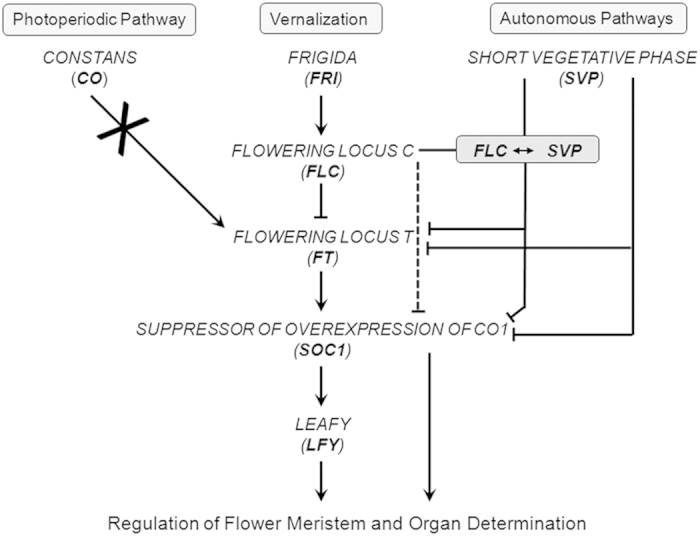
The hypothetical model of floral signal integration in apple. The model was proposed on the basis of available literature and expression analysis in the present study.

Since, the dormancy induction and its release in apple is controlled by low temperature^5^, the presence of low temperature responsive elements (C-repeat binding factor and low temperature responsive element) was also analyzed in promoter sequence of *MdDAMs*, which revealed presence of low temperature response elements (LTRE; Supplementary Table S4). The *MdMADS063* and *MdMADS125* have higher number of LTRE (two and seven, respectively) with in the 1.5Kb upstream region of transcription start site, which indicates their cold mediated regulation during dormancy period in apple. Similarly, presence of CBF in promoter regions of *DAMs* and their low temperature responsive expression was also reported in other plants^5^,^17^,^18^. Moreover, the expression of *MdMADS063* and *MdMADS125* was found to be induced in fruit tissues after which plant again undergoes next cycle of winter dormancy. Conclusively, the seasonal expression patterns of *DAM* genes in apple, peach, apricot, leafy spurge and poplar indicate that the orthologs of *SVP* have evolved to acquire the additional functions during seasonal dormancy in higher plant species beside their role in vegetative to reproductive transition and flower development, as reported in Arabidopsis. These results indicate the importance of *FLC*-like and *MdDAM* genes in regulating the dormancy. Therefore, we hypothesise that, *FLC-like* and *MdDAM* genes are the key regulators during dormant to active phase transition and their functions are largely conserved among higher dicotyledonous species. In addition, the expression of *LEAFY* (*LFY*), a downstream target of *SOC1*, was found to be increased along with SOC1 expression during dormancy release (Fig. 4I). Moreover, the photoperiodic pathway of flower initiation in Arabidopsis was found to be regulated by *CONSTANS* (*CO*), a non-MADS gene, however the expression pattern of CO orthologs in apple was not observed to be in accordance (Fig. 4H). This indicates the dormancy release in apple might not be under the control of photoperiod, which was also reported by Heide and Prestrud^5^. On the basis of present study and previous supporting literature, we hypothesized that the roles of apple orthologs of FLC, SVP, FRI, FT, SOC1 and LFY in dormancy and flower regulation are largely conserved, whereas CO does not seem to play role in photoperiodic floral signal integration (Fig. 5). However, functional characterization of these genes will reinforce the proposed hypothesis of winter dormancy and flowering time regulation.

The FPKM based expression of *MdMADS* genes obtained through RNA-seq was validated using qRT-PCR. The ten genes were randomly selected from various classes of type-II genes including *FLC*-like (*MdMADS135*), *DAM* (*MdMADS024*, *MdMADS125*), orthologs of *SOC1* (*MdMADS014*), *TM8* (*MdMADS111*), *AP3*/*PI* (*MdMADS044* and *MdMADS050*), *AGL15* (*MdMADS051*) *SEP* (*MdMADS123*) and gene of MIKC^C^-II clade (*MdMADS126*). Out of ten *MdMADS*, expression of six genes was observed in one or more of the tissues analyzed in present study. The relative expression analysis of *DAM* genes, *FLC*-like, orthologs of *SOC1*, *TM8*, *SEP* and *AP3*/*PI* was observed to be in accordance with FPKM based expression values (Supplementary Fig. S5).

**Figure 6:**
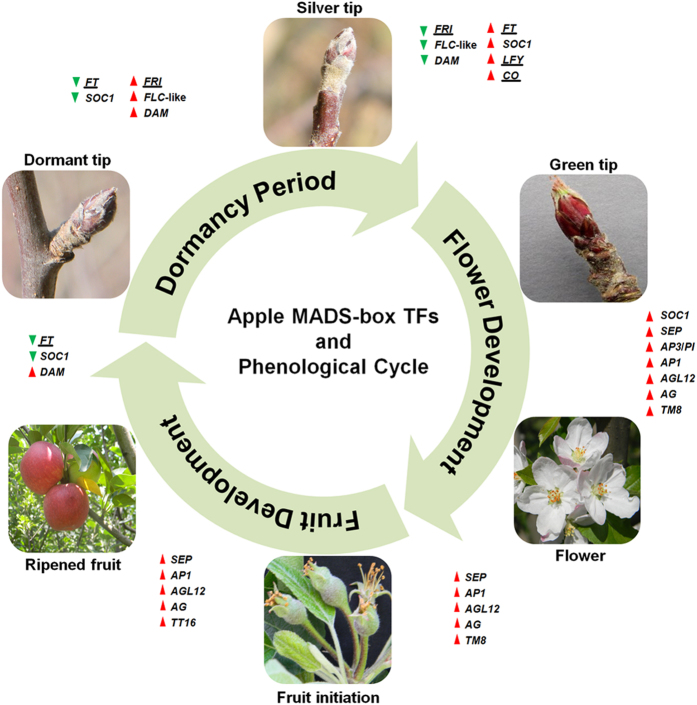
Pictorial representation of importance of *MdMADS-box* in various phenological events in apple. The complete phenological cycle of apple and the associated expression of *MdMADS-box* TFs of different classes are shown to be either up-regulated (red triangle) or down-regulated (green triangle). Few non-MADS genes (underlined) involved in dormancy and flowering time regulation are also shown.

In summary, the RNA-seq analysis revealed that expression of several *MdMADS* genes was modulated during dormancy to fruit ripening (Fig. 6). For instance, change in expression of *DAM*, *FLC*-like and orthologs of *SOC1* appear to be necessary from dormant to active phase transition, while the orthologs of *AG*, *SEP*, *AP3*/*PI*, *AGL12* and *TM8* are associated with flower development and fruit initiation. In addition to these genes, ortholog of *TT16* is also involved in fruit development. Thus, it is apparent that transitions of various developmental stages during apple phenology are associated with change in expression of *MdMADS* genes.

### Identification and network analysis of putative targets of orthologs of SVP in apple

In order to further evaluate the functional conservation of SVP orthologs in apple, a computational approach was used to determine the putative targets of MdDAM. Recently, downstream targets of Arabidopsis SVP protein were identified using Chip-seq approach^48^; using the same Chip-seq data, we identified 214 and 261 SVP binding motifs in vegetative and reproductive phase data of Arabidopsis, respectively. In order to identify the putative targets of MdDAM, the pattern search of these identified motifs against apple genome revealed the presence of 22 and 23 unique motifs of reproductive phase and vegetative phase, respectively (Supplementary Table S5). The protein orthologs of predicted apple target genes were searched in Arabidopsis, and these Arabidopsis proteins were further considered to build the respective protein-protein interaction (PPI) networks *viz.*, vegetative and reproductive phase PPI networks.

**Figure 7:**
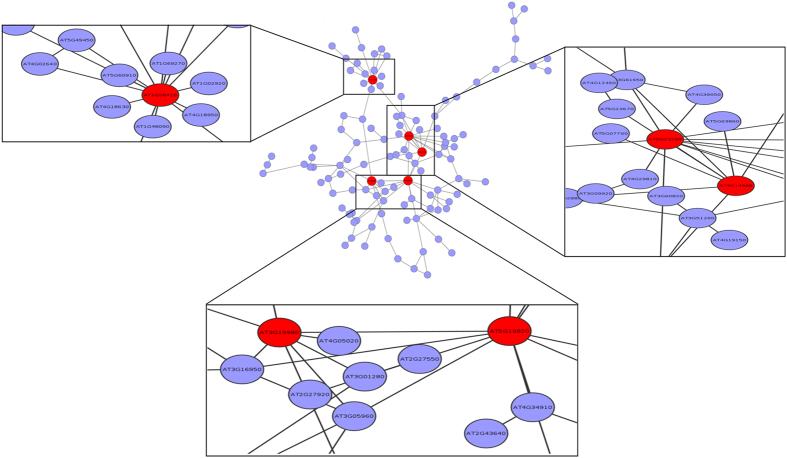
Protein-protein interaction network of Arabidopsis orthologs of putative targets of apple DAM transcription factors. The circles represent proteins, and the lines indicate an interaction between proteins. The highlighted Arabidopsis proteins represent five hub proteins *viz*, AT1G66410, AT5G23290, AT5G19820, AT3G19980 and AT4G14960. The interacting partners of each of the hub protein are also represented as zoomed-in view in squares.

Analysis of topology of interaction network of putative downstream targets of MdDAM protein revealed the presence of hub proteins. Compared to the PPI network of vegetative phase, the node degree distribution (probability distribution of degrees) of reproductive phase network approximated a power-law (Supplementary Fig. S6). The degree distribution results indicate the presence of few highly connected proteins in reproductive phase (Fig. 7), while isolated entities (not as a giant cluster) are present in vegetative phase network; therefore, we limited our analysis only to reproductive phase PPI network.

A total of five hubs (Supplementary Table S6) were obtained, which constituted ~4% of total proteins in largest connected component (Fig. 7). Further, the clusters of interacting proteins were annotated for their ontological characterization using agriGO^49^, implementing hyper-geometric test. The GO terms with false discovery rate (FDR) corrected p-values less than 0.05 were considered as significantly enriched for biological process and molecular function categories. In Arabidopsis, Gregis *et al.*^48^ reported that SVP was found to be involved in regulation of reproduction and flowering related pathways. In the present study, the similar inference was also deduced from gene ontology analysis, where majority of putative interactors were found to be involved in major signalling pathways: hormone and other stimulus, negative regulation of flower development and reproductive process, regulation of root meristem and seed development; that suggests the regulation of flowering control and other meristematic activities by SVP ortholog in apple (DAM) also (Supplementary Table S7). In addition, this analysis provides new insights towards the functional conservation and importance of SVP orthologs (DAM) in woody plants.

## Conclusions

Our analysis identified MADS-box TF family in apple genome, comprising of 142 proteins, which is second highest in number after soybean. The genome organization of apple *MdMADS* genes revealed that large number of *MdMADS* genes are present in clusters and significant expansion has taken place during recent whole genome duplication with equal contribution of segmental and tandem duplication. The phylogenetic analysis of MdMADS proteins with that of Arabidopsis and other dicotyledonous species resulted in identification of six DAM and four FLC-like proteins in apple. Using high-throughput Illumina RNA-seq approach, the expression of 63 *MdMADS* genes was detected in various developmental stages of different apple tissues. The expression profile of *DAM* and *FLC*-like genes and other genes (*FRI*, *FT*, *LFY*, *SOC1* and *CO*) was discussed in the light of previous supporting literature that helped us to assign putative functions to these genes during dormancy and flower time regulation in apple. In addition, computational approach was used to evaluate the functional conservation of SVP orthologs (DAM) in apple by identification and characterization of their putative downstream targets. Our findings are largely consistent with the previous studies in Arabidopsis. Overall, the computational and experimental approaches illustrated the importance of various members of MADS-box TFs family in diverse developmental processes throughout the phenological events in apple.

## Methods

### Data retrieval

The whole apple genome sequence data set used for the identification and annotation of MADS box proteins was downloaded from Phytozome (www.phytozome.net) and Apple GFDB (http://www.applegene.org). The MADS-box protein sequences of Arabidopsis were retrieved from TAIR10 (http://www.arabidopsis.org), while poplar, peach, and grape MADS-box protein sequences were retrieved from Phytozome v10.3 (http://www.phytozome.net). The cucumber MADS-box protein sequences were downloaded from Cucumber Genome Database (http://cucumber.genomics.org).

### Identification of MADS-box gene family in apple

The Hidden Markov Model (HMM) profile of MADS-box domain (PF00319) was retrieved from pfam v27.0^50^ and employed to identify the putative MADS-box proteins in apple genome. For this, “hmmsearch” of HMMER V3.0 was performed, with e-value 1e-04. The identified putative MADS proteins were then subjected to PfamScan program to confirm the presence of MADS domain and to identify additional domains with parameters: -e_seq 1e-04 -e_dom 1e-04 -clan_overlap. The orthologs of apple MADS-box were identified in *Arabidopsis thaliana* using BLASTP search against Arabidopsis proteome from TAIR 10 (http://www.arabidopsis.org). The putative MADS-box proteins were further classified into regular and non-regular proteins based on the similarity in BLASTP^51^ against non-redundant database with cut-off criterion of >50% similarity and >50% query coverage.

### Chromosomal mapping, nomenclature and gene duplication of MADS-box family

The coordinates of gene location on chromosome were extracted from general feature format (gff) file from phytozome and plotted on all 17 chromosomes using MapInspect program (http://www.plantbreeding.wur.nl/uk/software_mapinspect.html). Owing to the obscure nomenclature of apple MADS-box protein in NCBI and other databases, the identified putative apple MADS-box proteins were localized on apple chromosomes based on their genomic coordinates from 5′ to 3′ and numbered accordingly from top to bottom on 1 to 17 chromosomes along with suffix MdMADS to designate apple MADS-box protein (Supplementary Table S1). To analyze the duplication events in apple MADS-box gene family all-vs-all BLASTP^51^ was performed for 63,517 protein sequences with parameter: -evalue 1e-10, -outfmt 6, -num_threads 10 and -max_target_seqs 5. The BLASTP results were fed to MCScanX^52^ to identify the collinear gene pairs of MADS-box family in apple with default parameters.

### Phylogenetic analysis

The full length protein sequences of representative MADS-box TFs of Arabidopsis, poplar, peach, grape and cucumber were used to find the evolutionary relationship among MADS-box candidates in different species. The sequences of identified MADS-box proteins were aligned using ClustalW2 with default parameters. The multiple sequence alignment generated was then used to construct the phylogenetic tree by employing MEGA version 6.06 using neighbor-joining method with 1000 bootstrap replicates^53^.

### Identification of Putative targets of SVP like MADS protein in apple and their interaction network analysis

To identify the putative targets of orthologs of SVP (DAM) MADS-box proteins in apple, vegetative and reproductive phase chip-seq data of Arabidopsis SVP protein was used^48^. Further, all possible MADS-box nucleotide binding site patterns were extracted from AGRIS database (http://arabidopsis.med.ohio-state.edu). These nucleotide patterns were further searched against the Arabidopsis chromosomal coordinates identified through SVP chip-seq analysis in vegetative phase and reproductive phase separately; this was performed in order to limit the nucleotide patterns pertaining only to binding site of SVP protein. These identified patterns were searched against 2kb upstream sequence data of apple transcripts obtained from phytozome. The protein sequences of apple genes whose upstream region contains the SVP binding sites were extracted, and their orthologs were identified in Arabidopsis proteome dataset from TAIR 10. On the basis of BLASTP (e-value 1e-04), top hits were considered to build the protein-protein interaction (PPI) network.

We created two types of PPI networks: vegetative phase and reproductive phase PPI network. The networks were constructed by mapping Arabidopsis orthologs of apple candidate proteins onto the *Arabidopsis thaliana* Protein Interaction Network (AtPIN) interactome. AtPIN^54^ contains experimental and computational information pertaining to Arabidopsis PPIs. Proteins without any interaction partner were eliminated and the interactions that appeared more than once were simplified to single edges in the dataset. Degree distribution of both networks, *viz.* vegetative and reproductive was determined to compare their topological properties.

### Plant material

The plant material was collected from apple orchard located at Palchan, Manali, Himachal Pradesh, India (32° 18′ 36″ N, 77° 10′ 40″ E) during the year 2012–2013 of complete phenological cycle of apple. The 3 and 5 stages of dormant apical and spur buds, respectively, during the winter from November, 2012 to February, 2013; 6 stages of flower development in the month of April, 2013 and 7 stages of fruit development from May, 2013 to September, 2013 were collected. The collected samples were immediately frozen in liquid nitrogen and stored at −80 °C till further analysis.

### RNA-seq analysis

Total RNA was extracted from 100 mg of plant material using iRIS solution^55^. Total RNA was treated with RNase free DNase to eliminate genomic DNA contamination. The concentration of RNA was measured using NanoDrop UV-VIS spectrophotometer. The integrity of RNA was further checked on denaturing formaldehyde agarose gel. The RNA-Seq libraries were prepared using TruSeq RNA sample preparation kit v2 (Illumina Inc., USA) following manufacturer′s instructions. The libraries were quantified using Qubit dsDNA BR assay kit (Life technologies, USA) and insert size of libraries was determined using Agilent Bioanalyzer DNA 1000 chip (Agilent Technologies). For generating clusters, 10pM of each library was loaded onto the flow cell using TruSeq PE Cluster Kit v5 on cluster station (Illumina Inc., USA). The flow cell containing clonally amplified clusters was loaded onto the Genome Analyzer IIx (Illumina) and paired-end (PE) (2 × 72) sequencing was performed. The raw reads were quality filtered by means of NGS QC toolkit^56^ and the filtered high quality reads were then *de novo* assembled using CLC Genomics Wokbench 6.5 (http://www.clcbio.com). The assembled contigs were annotated by BLASTX search against non-redundant NCBI protein database with e-value cutoff 1e-05. The raw reads from Illumina GA IIx of all the analyzed samples were submitted as BioProject (PRJNA302879) to NCBI Sequence Read Archive under accession number SRP066478.

### Quantitative real-time PCR (qRT-PCR) analysis

First strand cDNA was synthesized from 1 μg of total RNA in 20 μl reaction using RevertAid RNAse H minus cDNA synthesis kit as per manufacturer’s instructions (Fermentas Life Sciences, USA). For qRT-PCR analysis, primers (Supplementary Table S8) were designed using Primer Express software version 3.0.1 (Invitrogen). The qRT-PCR assays were performed as described earlier^57^. For data normalization, GAPDH was used as internal control gene at different developmental stages^58^. The relative expression value was expressed as fold change calculated using comparative delta-delta Ct method^59^.

## Additional Information

**How to cite this article**: Kumar, G. *et al.* Comparative phylogenetic analysis and transcriptional profiling of MADS-box gene family identified *DAM* and *FLC*-like genes in apple (*Malus* x *domestica*). *Sci. Rep.* **6**, 20695; doi: 10.1038/srep20695 (2016).

## Supplementary Material

Supplementary Information
