# Supplementary material for: Comparative phylogenetic analysis and transcriptional profiling of MADS-box gene family identified DAM and FLC-like genes in apple (Malusx domestica)
Source: Sci Rep. 2016 Feb 9;6:20695. doi: 10.1038/srep20695 (PMC4746589; doi:10.1038/srep20695)
Supplement: Supplementary Information [file srep20695-s1.pdf]

Supplementary Information:

Comparative phylogenetic analysis and transcriptional profiling of MADS-box gene family identified *DAM* and *FLC*-like genes in apple (*Malus* x *domestica*)

Gulshan Kumar[1,2,†], Preeti Arya[1,2,†], Khushboo Gupta[1], Vinay Randhawa[1,2], Vishal Acharya[1,2], Anil Kumar Singh[1,2,*]

[1]Department of Biotechnology, CSIR-Institute of Himalayan Bioresource Technology, Palampur-176 061 (HP), India, [2]Academy of Scientific and Innovative Research, New Delhi, India

[†] These authors contribute equally to this work

*Correspondence and request for material should be addressed to AKS: (anil@ihbt.res.in; anils13@gmail.com)

Supplementary Information

Supplementary Figure S1. Chromosomal location and clustering of 142 MADS-box genes identified in apple. The chromosome number is indicated on the top of each chromosome. The color code represents the MdMADS belonging to different sub-families. The box indicates the 29 gene clusters on 17 apple chromosomes.

Supplementary Figure S2. Tandem and segmental duplication events in *MdMADS-box* gene family.

Supplementary Figure S3. Collinear genes pairs of *MdMADS-box* genes on all 17 apple chromosomes. Red lines indicate segmental duplication of *MdMADS-box* on 17 apple chromosomes, and grey lines represent collinear segments other than *MdMADS-box* genes.

Supplementary Figure S4. Phylogenetic tree of SVP orthologs of apple and other eudicots (Arabidopsis, peach, poplar, cucumber and grape).

Supplementary Figure S5. Relative expression of various MdMADS genes based upon FPKM based expression value and validated by quantitative reverse transcriptase-polymerase chain reaction (qRT-PCR). Pearson correlation coefficient between fold change FPKM and qRT-PCR was 0.34 (p-value=0.0005)

Supplementary Figure S6. Degree distributions of reproductive and vegetative phase network. The scatter plots show proteins (x-axis) versus the probability of a protein having *k* connections (y-axis). The reproductive phase network indicates scale-free nature.

Supplementary Table S1. List of MdMADS, their annotation and orthologs in Arabidopsis.

Supplementary Table S2. Comparison of MdMADS identified in present study with that of MdMADS identified by Tian et al. (2015).

Supplementary Table S3. The sample abbreviation and their collection intervals.

Supplementary Table S4. List of low temperature responsive elements in 1.5Kb upstream promoter regions of MdDAMs.

Supplementary Table S5. List of DNA binding site sequence for MdMADS proteins identified in apple.

Supplementary Table S6. List of hub proteins and their interacting partners in Arabidopsis identified through protein-protein interaction network and their orthologs in apple.

Supplementary Table S7. Over-represented biological process and molecular function gene ontology terms associated with reproductive phase network proteins.

Supplementary Table S8. List of primer sequence of randomly selected 10 MdMADS genes.

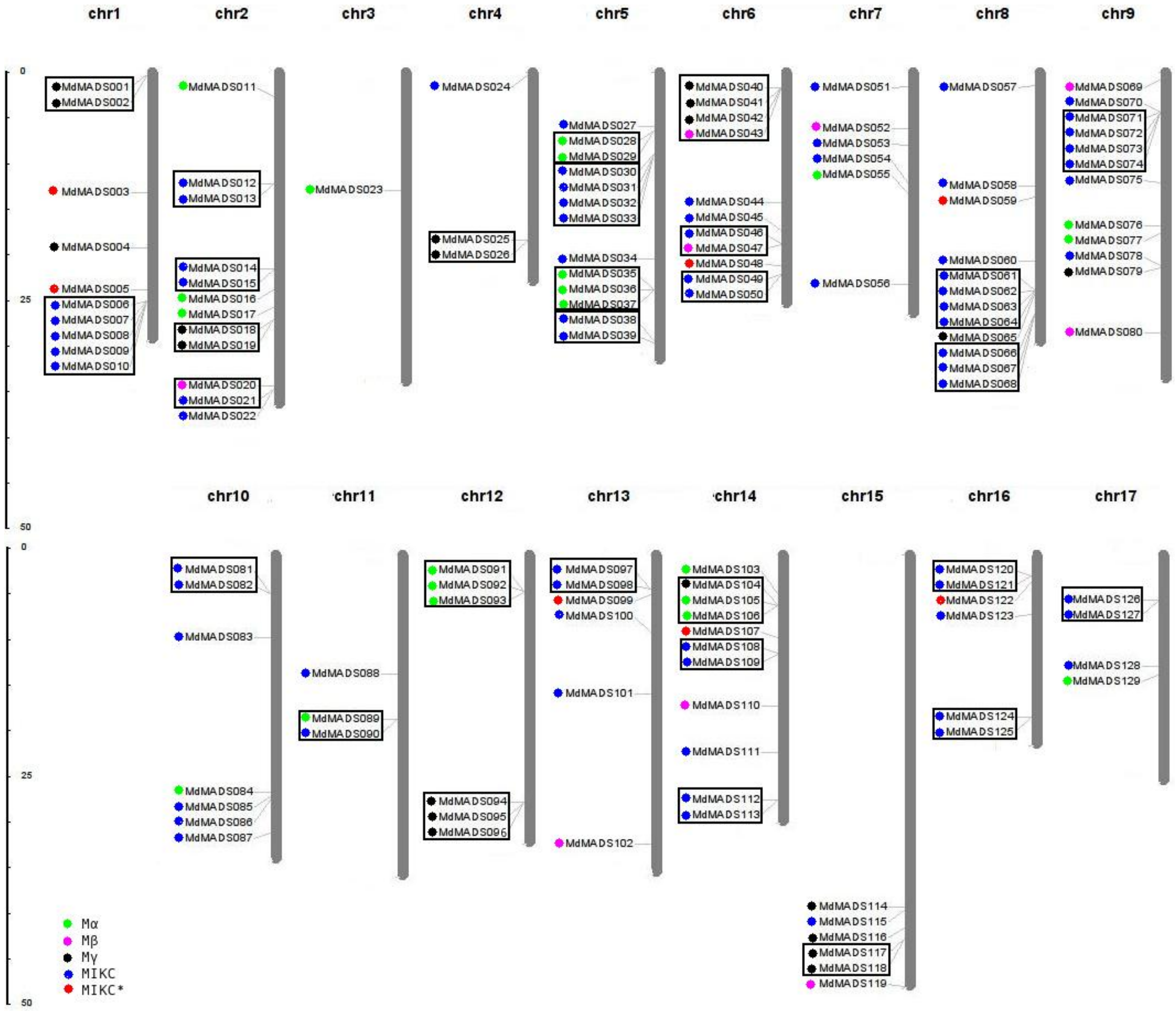

Supplementary Figure S1. Chromosomal location and clustering of 142 MADS-box genes identified in apple. The chromosome number is indicated on the top of each chromosome. The color code represents the MdMADS belonging to different sub-families. The box indicates the 29 gene clusters on 17 apple chromosomes.

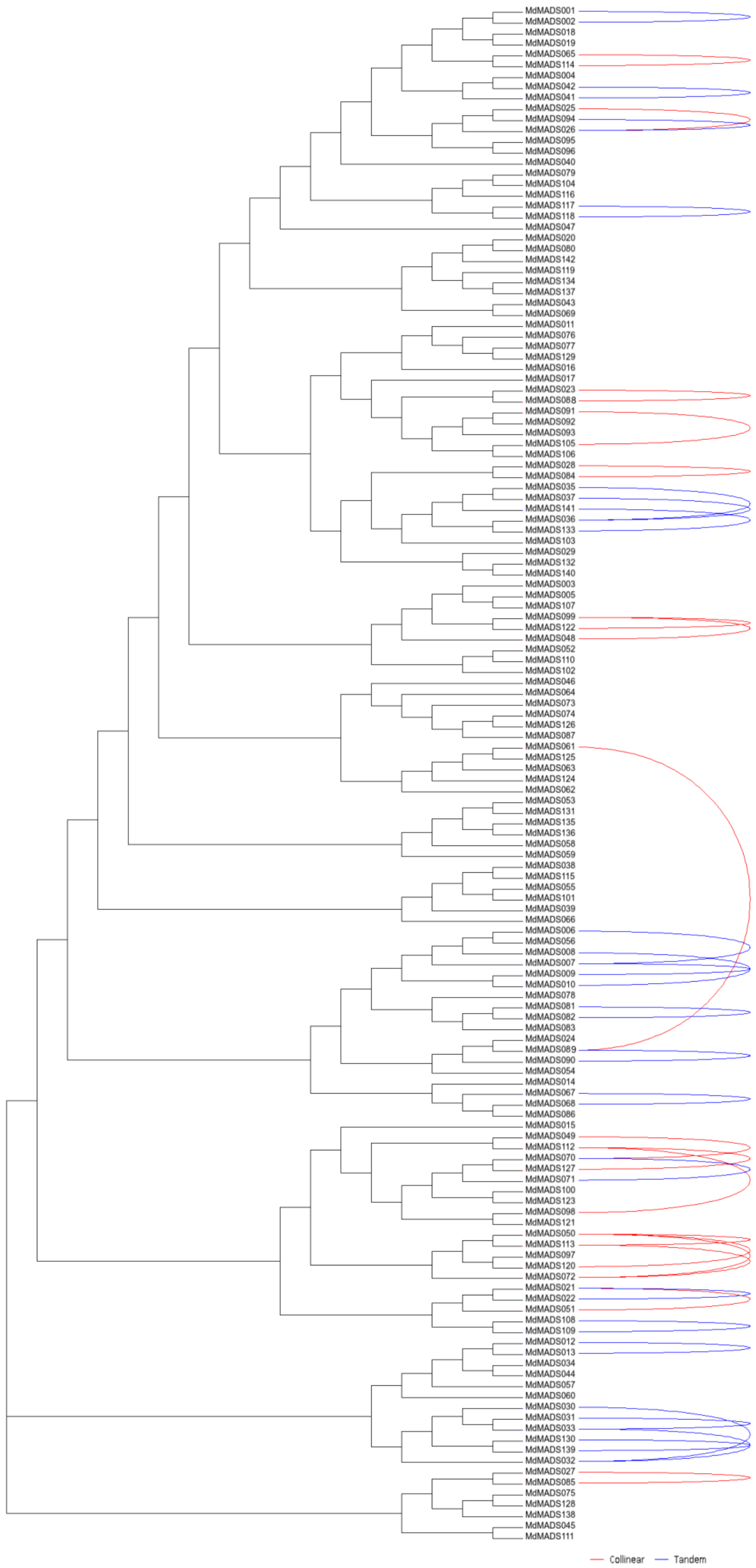

Supplementary Figure S2. Tandem and segmental duplication events in *MdMADS-box* gene family.

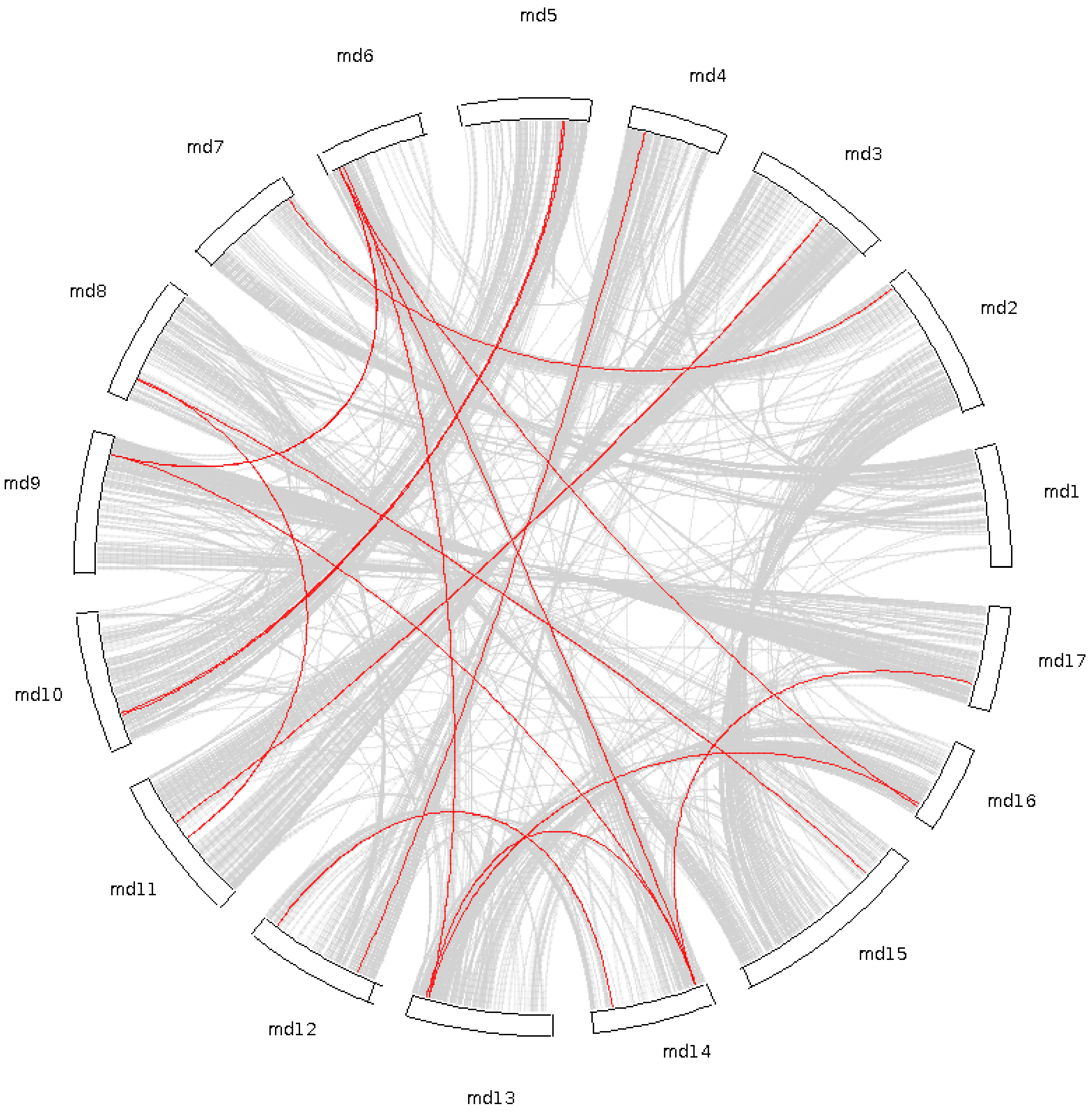

Supplementary Figure S3. Collinear genes pairs of *MdMADS-box* genes on all 17 apple chromosomes. Red lines indicate segmental duplication of *MdMADS-box* on 17 apple chromosomes, and grey lines represent collinear segments other than *MdMADS-box* genes.

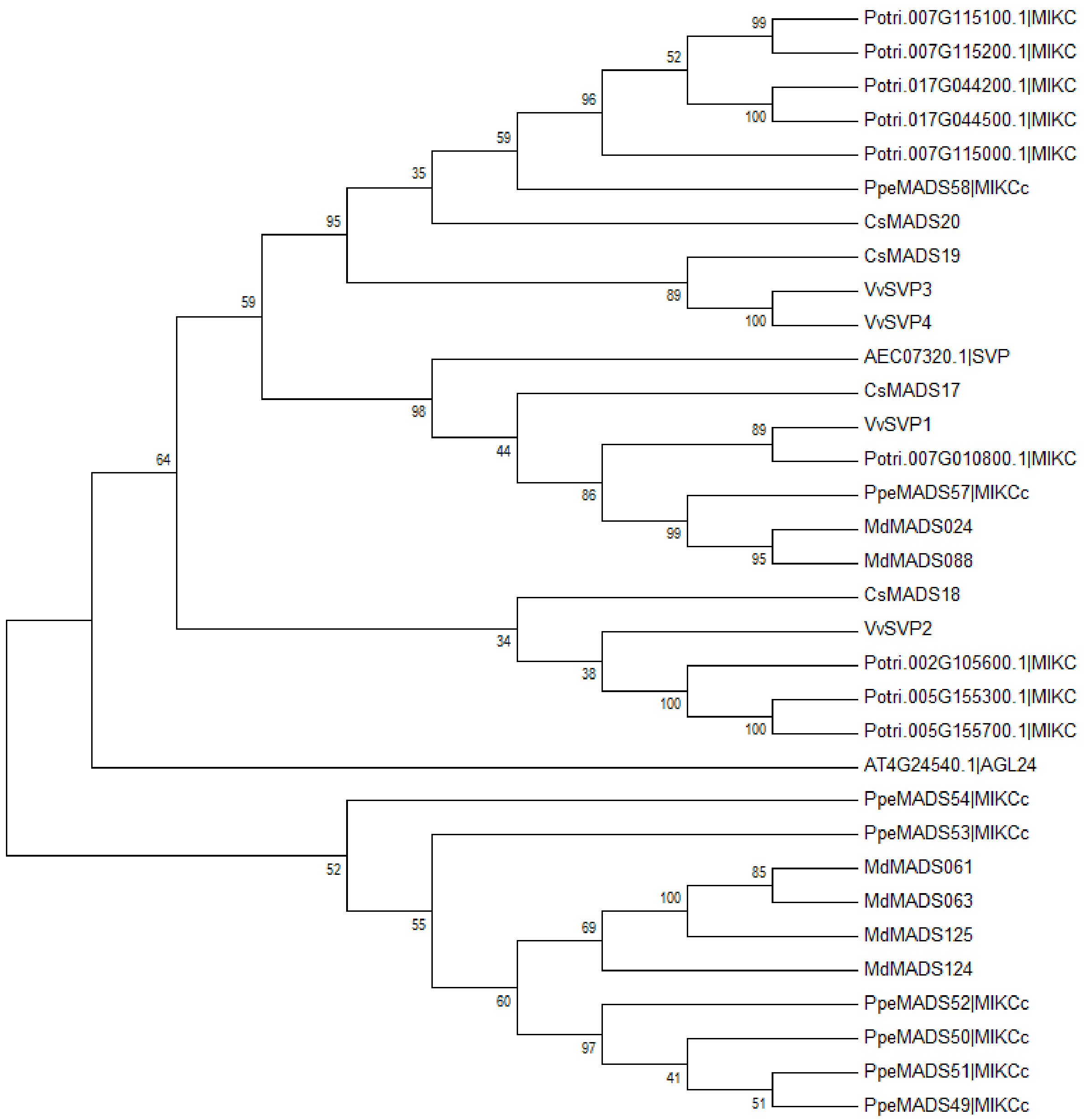

Supplementary Figure S4. Phylogenetic tree of SVP orthologs of apple and other eudicots (Arabidopsis, peach, poplar, cucumber and grape).

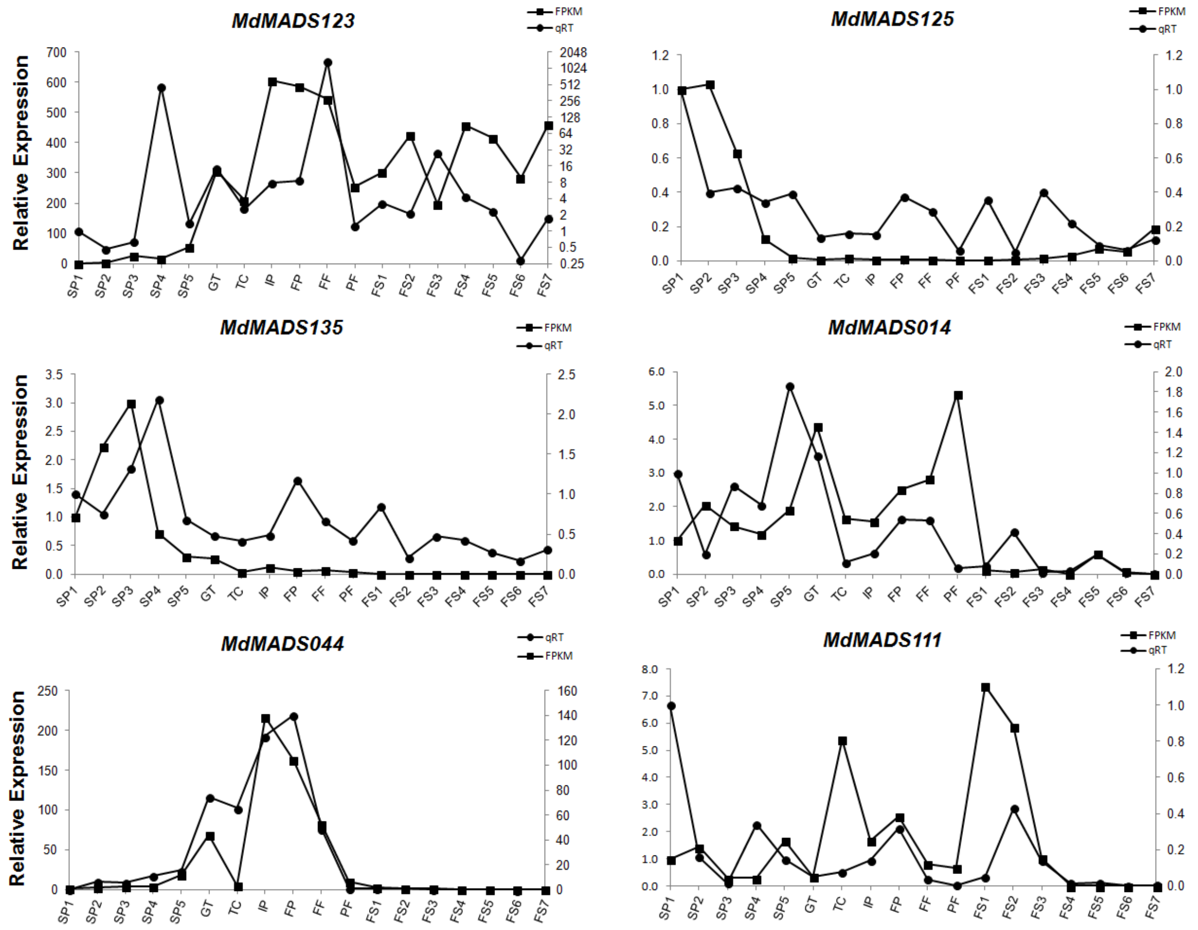

Supplementary Figure S5. Relative expression of various MdMADS genes based upon FPKM based expression value and validated by quantitative reverse transcriptase-polymerase chain reaction (qRT-PCR). Pearson correlation coefficient between fold change FPKM and qRT-PCR was 0.34 (p-value=0.0005)

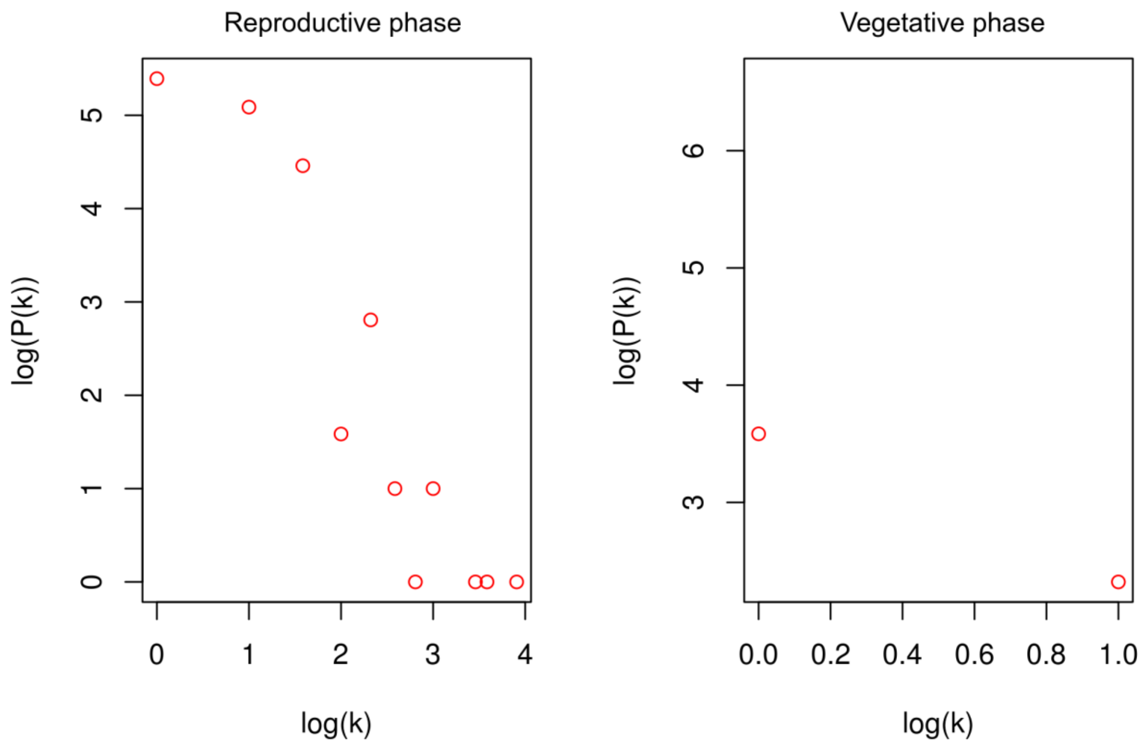

Supplementary Figure S6. Degree distributions of reproductive and vegetative phase network. The scatter plots show proteins (x-axis) versus the probability of a protein having *k* connections (y-axis). The reproductive phase network indicates scale-free nature.

Supplementary Table S1. List of MdMADS, their annotation and orthologs in Arabidopsis.

Gene	Protein	protein identifier	RNA-seq contig	Chromosome no.	CDS length	Protein length	Strand	Regular / Non-regular	Type	Duplication	At Ortholog	Score	E-value
MdMADS001	MdMADS001	MDP0000152718	No hit	chr01	471	156	+	Regular	Mγ	Tandem	AT5G48670.1	104	2.00E-23
MdMADS002	MdMADS002	MDP0000159587	No hit	chr01	471	156	+	Regular	Mγ	Tandem	AT5G48670.1	104	2.00E-23
MdMADS003	MdMADS003	MDP0000146415	Contig_49861	chr01	705	234	-	Non-regular	MIKC*	Dispersed	AT2G03060.2	108	2.00E-24
MdMADS004	MdMADS004	MDP0000133671	No hit	chr01	723	240	+	Regular	Mγ	Dispersed	AT5G48670.1	142	2.00E-34
MdMADS005	MdMADS005	MDP0000919323	No hit	chr01	303	101	-	Regular	MIKC*	Dispersed	AT2G03060.2	107	2.00E-24
MdMADS006	MdMADS006	MDP0000144597	Contig_13746	chr01	777	258	+	Regular	MIKC^{C}	Segmental	AT5G62165.1	187	9.00E-48
MdMADS007	MdMADS007	MDP0000462145	No hit	chr01	597	199	-	Regular	MIKC^{C}	Tandem	AT5G62165.1	139	2.00E-33
MdMADS008	MdMADS008	MDP0000249592	Contig_8881	chr01	675	224	-	Regular	MIKC^{C}	Tandem	AT5G62165.4	179	1.00E-49
MdMADS009	MdMADS009	MDP0000449389	No hit	chr01	1254	418	+	Regular	MIKC^{C}	Segmental	AT5G51890.1	360	e-100
MdMADS010	MdMADS010	MDP0000200272	No hit	chr01	237	79	+	Regular	MIKC^{C}	Tandem	AT5G62165.1	111	1.00E-25
MdMADS011	MdMADS011	MDP0000696331	Contig_40581	chr02	579	193	-	Regular	Mα	Dispersed	AT2G24840.1	47	9.00E-06
MdMADS012	MdMADS012	MDP0000315938	No hit	chr02	699	232	+	Regular	MIKC^{C}	Tandem	AT3G54340.1	194	4.00E-50
MdMADS013	MdMADS013	MDP0000184294	Contig_25797	chr02	699	232	+	Regular	MIKC^{C}	Tandem	AT3G54340.1	194	4.00E-50
MdMADS014	MdMADS014	MDP0000314765	Contig_30208	chr02	759	252	+	Regular	MIKC^{C}	Dispersed	AT2G45660.1	248	3.00E-66
MdMADS015	MdMADS015	MDP0000149676	Contig_47159	chr02	636	212	+	Regular	MIKC^{C}	Dispersed	AT2G45650.1	121	3.00E-28
MdMADS016	MdMADS016	MDP0000237091	No hit	chr02	705	234	-	Regular	Mα	Dispersed	AT5G60440.1	104	4.00E-23
MdMADS017	MdMADS017	MDP0000162019	No hit	chr02	1614	537	-	Non-regular	Mα	Dispersed	AT2G24840.1	76	5.00E-14
MdMADS018	MdMADS018	MDP0000123878	No hit	chr02	387	128	+	Regular	Mγ	Proximal	AT5G48670.1	70	4.00E-13
MdMADS019	MdMADS019	MDP0000726295	No hit	chr02	387	129	-	Regular	Mγ	Proximal	AT5G48670.1	74	2.00E-14
MdMADS020	MdMADS020	MDP0000309181	Contig_220	chr02	1923	640	-	Regular	Mβ	Segmental	AT2G41540.1	353	2.00E-97
MdMADS021	MdMADS021	MDP0000156783	Contig_35108	chr02	1017	338	-	Regular	MIKC^{C}	Segmental	AT5G13790.1	186	2.00E-47
MdMADS022	MdMADS022	MDP0000136650	Contig_35108	chr02	1017	338	-	Regular	MIKC^{C}	Tandem	AT5G13790.1	167	8.00E-42
MdMADS023	MdMADS023	MDP0000155389	Contig_56230	chr03	663	220	-	Regular	Mα	Segmental	AT2G24840.1	106	1.00E-23
MdMADS024	MdMADS024	MDP0000209705	Contig_34137	chr04	687	228	+	Regular	MIKC^{C}	Dispersed	AT2G22540.1	261	3.00E-70
MdMADS025	MdMADS025	MDP0000212925	Contig_64541	chr04	618	205	+	Regular	Mγ	Segmental	AT5G48670.1	105	2.00E-23
MdMADS026	MdMADS026	MDP0000129298	No hit	chr04	678	225	+	Non-regular	Mγ	Proximal	AT5G48670.1	122	2.00E-28
MdMADS027	MdMADS027	MDP0000250080	Contig_35532	chr05	696	231	-	Regular	MIKC^{C}	Segmental	AT4G18960.1	290	4.00E-79
MdMADS028	MdMADS028	MDP0000120467	No hit	chr05	528	175	-	Regular	Mα	Segmental	AT2G34440.1	125	1.00E-29
MdMADS029	MdMADS029	MDP0000696333	No hit	chr05	636	212	-	Regular	Mα	Dispersed	AT5G60440.1	129	1.00E-30
MdMADS030	MdMADS030	MDP0000183226	No hit	chr05	597	199	-	Regular	MIKC^{C}	Tandem	AT5G23260.2	155	1.00E-38

MdMADS031	MdMADS031	MDP0000181583	Contig_57607	chr05	960	319	-	Regular	$MIKC^C$	Tandem	AT5G23260.2	185	3.00E-47
MdMADS032	MdMADS032	MDP0000179718	No hit	chr05	513	171	-	Regular	$MIKC^C$	Tandem	AT5G23260.2	106	7.00E-24
MdMADS033	MdMADS033	MDP0000234073	No hit	chr05	576	192	-	Regular	$MIKC^C$	Tandem	AT5G23260.2	156	9.00E-39
MdMADS034	MdMADS034	MDP0000224779	No hit	chr05	723	240	-	Regular	$MIKC^C$	Dispersed	AT3G54340.1	200	6.00E-52
MdMADS035	MdMADS035	MDP0000233925	No hit	chr05	648	215	+	Regular	Mα	Tandem	AT5G60440.1	209	2.00E-54
MdMADS036	MdMADS036	MDP0000233926	No hit	chr05	408	135	+	Regular	Mα	Tandem	AT2G24840.1	99	6.00E-22
MdMADS037	MdMADS037	MDP0000278770	No hit	chr05	1008	335	-	Regular	Mα	Tandem	AT5G60440.1	225	3.00E-59
MdMADS038	MdMADS038	MDP0000228942	No hit	chr05	324	107	+	Regular	$MIKC^C$	Proximal	AT2G14210.1	119	5.00E-28
MdMADS039	MdMADS039	MDP0000475549	No hit	chr05	435	145	+	Regular	$MIKC^C$	Proximal	AT4G37940.1	152	1.00E-37
MdMADS040	MdMADS040	MDP0000451827	Contig_63558	chr06	822	274	+	Non-regular	Mγ	Proximal	AT5G48670.1	89	3.00E-18
MdMADS041	MdMADS041	MDP0000299637	No hit	chr06	645	214	+	Regular	Mγ	Tandem	AT5G48670.1	141	3.00E-34
MdMADS042	MdMADS042	MDP0000236438	No hit	chr06	612	203	-	Regular	Mγ	Tandem	AT5G48670.1	135	2.00E-32
MdMADS043	MdMADS043	MDP0000664841	No hit	chr06	1071	357	-	Regular	Mβ	Dispersed	AT5G55690.1	72	4.00E-13
MdMADS044	MdMADS044	MDP0000133037	Contig_33505	chr06	723	240	+	Regular	$MIKC^C$	Dispersed	AT3G54340.1	200	6.00E-52
MdMADS045	MdMADS045	MDP0000312382	Contig_22559	chr06	1590	529	+	Regular	$MIKC^C$	Segmental	AT3G48680.1	378	e-105
MdMADS046	MdMADS046	MDP0000132226	No hit	chr06	429	143	-	Regular	$MIKC^C$	Dispersed	AT4G24540.1	82	8.00E-17
MdMADS047	MdMADS047	MDP0000670559	No hit	chr06	1002	334	-	Non-regular	Mβ	Singleton	AT5G65330.1	55	5.00E-08
MdMADS048	MdMADS048	MDP0000193129	Contig_41493	chr06	2226	741	-	Regular	MIKC*	Segmental	AT1G18750.1	299	6.00E-81
MdMADS049	MdMADS049	MDP0000574222	Contig_48525	chr06	564	188	+	Regular	$MIKC^C$	Segmental	AT2G03710.2	163	5.00E-41
MdMADS050	MdMADS050	MDP0000218020	Contig_61394	chr06	900	299	-	Regular	$MIKC^C$	Segmental	AT5G60910.1	243	1.00E-64
MdMADS051	MdMADS051	MDP0000254052	Contig_35107	chr07	810	269	-	Regular	$MIKC^C$	Segmental	AT5G13790.1	214	6.00E-56
MdMADS052	MdMADS052	MDP0000216605	No hit	chr07	1019	339	-	Non-regular	Mβ	Dispersed	AT2G39970.1	56	3.00E-08
MdMADS053	MdMADS053	MDP0000127771	Contig_8136	chr07	312	103	+	Regular	$MIKC^C$	Dispersed	AT2G03710.3	61	1.00E-10
MdMADS054	MdMADS054	MDP0000148857	Contig_8675	chr07	492	163	+	Regular	$MIKC^C$	Dispersed	AT2G45660.1	120	5.00E-28
MdMADS055	MdMADS055	MDP0000783966	No hit	chr07	264	88	+	Regular	Mα	Dispersed	AT4G37940.1	116	3.00E-27
MdMADS056	MdMADS056	MDP0000060753	Contig_19842	chr07	591	197	+	Regular	$MIKC^C$	Proximal	AT5G62165.1	205	1.00E-53
MdMADS057	MdMADS057	MDP0000220168	No hit	chr08	657	218	+	Regular	$MIKC^C$	Dispersed	AT3G54340.1	213	5.00E-56
MdMADS058	MdMADS058	MDP0000152821	Contig_40010	chr08	1437	478	+	Regular	$MIKC^C$	Dispersed	AT1G22130.1	104	2.00E-22
MdMADS059	MdMADS059	MDP0000243965	Contig_59418	chr08	1044	347	-	Regular	MIKC*	Dispersed	AT1G22130.1	225	4.00E-59
MdMADS060	MdMADS060	MDP0000286643	No hit	chr08	762	253	+	Regular	$MIKC^C$	Dispersed	AT5G20240.1	241	2.00E-64
MdMADS061	MdMADS061	MDP0000259294	No hit	chr08	786	261	+	Regular	$MIKC^C$	Segmental	AT4G24540.1	101	4.00E-22
MdMADS062	MdMADS062	MDP0000259296	No hit	chr08	372	123	+	Regular	$MIKC^C$	Proximal	AT4G24540.1	94	2.00E-20
MdMADS063	MdMADS063	MDP0000527190	Contig_2275	chr08	870	290	-	Regular	$MIKC^C$	Proximal	AT4G24540.1	114	8.00E-26

MdMADS064	MdMADS064	MDP0000378412	No hit	chr08	516	172	-	Regular	$MIKC^C$	Proximal	AT4G24540.1	97	7.00E-21
MdMADS065	MdMADS065	MDP0000925348	No hit	chr08	768	256	-	Regular	Mγ	Segmental	AT5G48670.1	202	1.00E-52
MdMADS066	MdMADS066	MDP0000315764	Contig_22333	chr08	1925	640	-	Non-regular	$MIKC^C$	Segmental	AT1G33780.1	351	8.00E-97
MdMADS067	MdMADS067	MDP0000193953	No hit	chr08	309	103	-	Regular	$MIKC^C$	Tandem	AT1G71692.1	122	4.00E-29
MdMADS068	MdMADS068	MDP0000192806	No hit	chr08	309	103	-	Regular	$MIKC^C$	Tandem	AT1G71692.1	122	4.00E-29
MdMADS069	MdMADS069	MDP0000296123	No hit	chr09	1026	341	-	Regular	Mβ	Dispersed	AT5G55690.1	64	2.00E-10
MdMADS070	MdMADS070	MDP0000289858	Contig_39835	chr09	654	217	-	Regular	$MIKC^C$	Segmental	AT3G02310.1	293	8.00E-80
MdMADS071	MdMADS071	MDP0000605482	No hit	chr09	450	150	+	Regular	$MIKC^C$	Tandem	AT5G15800.1	245	7.00E-66
MdMADS072	MdMADS072	MDP0000132738	Contig_37268	chr09	312	103	-	Regular	$MIKC^C$	Segmental	AT5G60910.1	115	5.00E-27
MdMADS073	MdMADS073	MDP0000143531	No hit	chr09	357	118	-	Regular	$MIKC^C$	Proximal	AT4G24540.1	84	1.00E-17
MdMADS074	MdMADS074	MDP0000223851	No hit	chr09	315	104	+	Regular	$MIKC^C$	Proximal	AT4G24540.1	80	4.00E-16
MdMADS075	MdMADS075	MDP0000324259	Contig_9560	chr09	727	243	-	Regular	$MIKC^C$	Tandem	AT3G58780.1	335	1.00E-92
MdMADS076	MdMADS076	MDP0000611204	No hit	chr09	756	252	+	Non-regular	Mα	Dispersed	AT2G22540.1	70	2.00E-12
MdMADS077	MdMADS077	MDP0000291070	Contig_4705	chr09	1782	593	+	Non-regular	Mα	Tandem	AT1G17350.1	268	7.00E-72
MdMADS078	MdMADS078	MDP0000518032	Contig_27449	chr09	321	107	-	Regular	$MIKC^C$	Dispersed	AT4G22950.1	80	2.00E-16
MdMADS079	MdMADS079	MDP0000254632	No hit	chr09	1320	439	-	Non-regular	Mγ	Dispersed	AT5G26630.1	102	8.00E-22
MdMADS080	MdMADS080	MDP0000123808	No hit	chr09	612	203	-	Regular	Mβ	Proximal	AT5G55690.1	55	4.00E-08
MdMADS081	MdMADS081	MDP0000270062	Contig_10606	chr10	1173	390	-	Regular	$MIKC^C$	Tandem	AT4G22950.1	189	3.00E-48
MdMADS082	MdMADS082	MDP0000248130	Contig_30632	chr10	540	179	+	Non-regular	$MIKC^C$	Tandem	AT4G11880.1	115	1.00E-26
MdMADS083	MdMADS083	MDP0000205637	No hit	chr10	792	263	-	Non-regular	$MIKC^C$	Tandem	AT5G23150.1	113	1.00E-25
MdMADS084	MdMADS084	MDP0000274606	No hit	chr10	543	180	+	Regular	Mα	Segmental	AT2G34440.1	127	6.00E-30
MdMADS085	MdMADS085	MDP0000324166	No hit	chr10	730	244	-	Regular	$MIKC^C$	Segmental	AT4G18960.1	356	8.00E-99
MdMADS086	MdMADS086	MDP0000316609	Contig_48693	chr10	720	239	+	Regular	$MIKC^C$	Proximal	AT1G71692.1	219	9.00E-58
MdMADS087	MdMADS087	MDP0000311107	Contig_62351	chr10	1164	387	+	Non-regular	$MIKC^C$	Dispersed	AT4G24540.1	73	4.00E-13
MdMADS088	MdMADS088	MDP0000233948	Contig_40529	chr11	765	254	+	Regular	$MIKC^C$	Segmental	AT2G22540.1	102	2.00E-53
MdMADS089	MdMADS089	MDP0000314058	No hit	chr11	1707	568	+	Non-regular	Mα	Segmental	AT2G24840.1	205	9.00E-22
MdMADS090	MdMADS090	MDP0000233843	Contig_24584	chr11	182	61	+	Regular	$MIKC^C$	Tandem	AT2G22540.1	112	4.00E-26
MdMADS091	MdMADS091	MDP0000523644	No hit	chr12	579	193	-	Regular	Mα	Segmental	AT5G60440.1	90	9.00E-19
MdMADS092	MdMADS092	MDP0000713024	No hit	chr12	606	202	-	Non-regular	Mα	Proximal	AT5G60440.1	90	1.00E-18
MdMADS093	MdMADS093	MDP0000232338	No hit	chr12	1008	335	-	Non-regular	Mα	Proximal	AT2G24840.1	78	8.00E-15
MdMADS094	MdMADS094	MDP0000307605	No hit	chr12	639	212	-	Regular	Mγ	Segmental	AT5G48670.1	117	6.00E-27
MdMADS095	MdMADS095	MDP0000834768	No hit	chr12	231	77	+	Regular	Mγ	Segmental	AT5G48670.1	78	1.00E-15
MdMADS096	MdMADS096	MDP0000599449	No hit	chr12	678	226	-	Regular	Mγ	Proximal	AT5G48670.1	115	2.00E-26
MdMADS097	MdMADS097	MDP0000269921	Contig_42886	chr13	624	207	-	Regular	$MIKC^C$	Proximal	AT1G69120.1	271	2.00E-73

MdMADS098	MdMADS098	MDP0000326906	No hit	chr13	692	231	+	Regular	$MIKC^{C}$	Segmental	AT2G03710.2	241	3.00E-64
MdMADS099	MdMADS099	MDP0000146231	Contig_38809	chr13	1212	403	+	Regular	MIKC*	Segmental	AT2G03060.2	278	6.00E-75
MdMADS100	MdMADS100	MDP0000370413	No hit	chr13	471	157	-	Regular	$MIKC^{C}$	Dispersed	AT1G24260.1	259	5.00E-70
MdMADS101	MdMADS101	MDP0000285581	No hit	chr13	735	244	+	Non-regular	$MIKC^{C}$	Dispersed	AT4G37940.1	114	7.00E-26
MdMADS102	MdMADS102	MDP0000229755	Contig_4976	chr13	966	321	+	Non-regular	Mβ	Dispersed	AT2G39970.1	80	1.00E-15
MdMADS103	MdMADS103	MDP0000221590	Contig_2967	chr14	1065	354	-	Regular	Mα	Dispersed	AT5G60440.1	155	3.00E-38
MdMADS104	MdMADS104	MDP0000296791	No hit	chr14	771	256	-	Non-regular	Mγ	Dispersed	AT2G40210.1	101	5.00E-22
MdMADS105	MdMADS105	MDP0000287530	No hit	chr14	399	132	-	Regular	Mα	Segmental	AT5G60440.1	73	5.00E-14
MdMADS106	MdMADS106	MDP0000289392	No hit	chr14	441	146	-	Regular	Mα	Proximal	AT5G60440.1	57	4.00E-09
MdMADS107	MdMADS107	MDP0000773068	No hit	chr14	264	88	-	Regular	MIKC*	Dispersed	AT2G03060.2	108	1.00E-24
MdMADS108	MdMADS108	MDP0000135652	Contig_53993	chr14	603	200	+	Regular	$MIKC^{C}$	Tandem	AT3G57390.1	148	2.00E-36
MdMADS109	MdMADS109	MDP0000144516	Contig_53993	chr14	801	266	-	Regular	$MIKC^{C}$	Tandem	AT3G57390.1	160	1.00E-39
MdMADS110	MdMADS110	MDP0000322264	Contig_2034	chr14	810	269	+	Non-regular	Mβ	Dispersed	AT2G39970.1	90	1.00E-18
MdMADS111	MdMADS111	MDP0000300752	Contig_29003	chr14	636	211	-	Regular	$MIKC^{C}$	Dispersed	AT4G09960.2	150	4.00E-37
MdMADS112	MdMADS112	MDP0000326390	Contig_29027	chr14	745	249	+	Regular	$MIKC^{C}$	Segmental	AT3G02310.1	288	2.00E-78
MdMADS113	MdMADS113	MDP0000289836	Contig_42405	chr14	861	286	-	Regular	$MIKC^{C}$	Segmental	AT5G60910.1	270	9.00E-73
MdMADS114	MdMADS114	MDP0000846004	No hit	chr15	765	255	+	Regular	Mγ	Segmental	AT5G48670.1	184	4.00E-47
MdMADS115	MdMADS115	MDP0000234911	No hit	chr15	429	142	+	Regular	$MIKC^{C}$	Dispersed	AT2G14210.1	119	5.00E-28
MdMADS116	MdMADS116	MDP0000134211	No hit	chr15	267	88	+	Regular	Mγ	Singleton	AT2G40210.1	42	9.00E-05
MdMADS117	MdMADS117	MDP0000186886	Contig_33310	chr15	813	270	+	Regular	Mγ	Tandem	AT5G48670.1	84	8.00E-17
MdMADS118	MdMADS118	MDP0000192342	No hit	chr15	552	183	+	Regular	Mγ	Tandem	AT1G22590.2	83	8.00E-17
MdMADS119	MdMADS119	MDP0000303501	No hit	chr15	927	308	+	Non-regular	Mβ	Dispersed	AT5G55690.1	62	4.00E-10
MdMADS120	MdMADS120	MDP0000013331	Contig_32487	chr16	714	238	-	Regular	$MIKC^{C}$	Segmental	AT1G69120.1	299	9.00E-82
MdMADS121	MdMADS121	MDP0000280009	Contig_36698	chr16	786	261	-	Regular	$MIKC^{C}$	Segmental	AT5G15800.1	240	8.00E-64
MdMADS122	MdMADS122	MDP0000147667	Contig_41802	chr16	1419	472	-	Regular	MIKC*	Segmental	AT2G03060.2	265	6.00E-71
MdMADS123	MdMADS123	MDP0000220008	Contig_41430	chr16	858	285	+	Regular	$MIKC^{C}$	Segmental	AT1G24260.2	300	7.00E-82
MdMADS124	MdMADS124	MDP0000255146	No hit	chr16	924	307	-	Regular	$MIKC^{C}$	Proximal	AT4G24540.1	97	2.00E-20
MdMADS125	MdMADS125	MDP0000322567	Contig_168	chr16	741	246	-	Regular	$MIKC^{C}$	Proximal	AT4G24540.1	114	6.00E-26
MdMADS126	MdMADS126	MDP0000277582	Contig_10477	chr17	738	245	+	Non-regular	$MIKC^{C}$	Dispersed	AT4G24540.1	86	2.00E-17
MdMADS127	MdMADS127	MDP0000366022	Contig_49417	chr17	757	253	+	Regular	$MIKC^{C}$	Segmental	AT3G02310.1	332	2.00E-91
MdMADS128	MdMADS128	MDP0000242009	Contig_41021	chr17	783	260	-	Regular	$MIKC^{C}$	Segmental	AT3G58780.3	292	2.00E-79
MdMADS129	MdMADS129	MDP0000315688	No hit	chr17	750	249	+	Regular	Mα	Dispersed	AT5G60440.1	75	3.00E-14
MdMADS130	MdMADS130	MDP0000128016	No hit	Unanchored	420	140	-	Regular	$MIKC^{C}$	Tandem	AT5G23260.2	152	6.00E-38
MdMADS131	MdMADS131	MDP0000138447	No hit	unanchored	312	103	-	Regular	$MIKC^{C}$	Dispersed	AT1G24260.2	57	2.00E-09

MdMADS132	MdMADS132	MDP0000159062	No hit	unanchored	612	203	+	Regular	Mα	Proximal	AT2G24840.1	110	6.00E-25
MdMADS133	MdMADS133	MDP0000168914	No hit	unanchored	714	237	-	Regular	Mα	Tandem	AT5G60440.1	179	1.00E-45
MdMADS134	MdMADS134	MDP0000185853	No hit	unanchored	948	315	+	Regular	Mβ	Dispersed	AT5G55690.1	69	4.00E-12
MdMADS135	MdMADS135	MDP0000186558	Contig_28	unanchored	648	215	+	Non-regular	$MIKC^C$	Proximal	AT2G03710.2	98	4.00E-21
MdMADS136	MdMADS136	MDP0000218227	Contig_2858	unanchored	279	92	+	Regular	$MIKC^C$	Proximal	AT5G15800.1	96	5.00E-21
MdMADS137	MdMADS137	MDP0000251651	Contig_19389	unanchored	1594	530	-	Non-regular	Mβ	Proximal	AT1G58110.1	149	5.00E-36
MdMADS138	MdMADS138	MDP0000268317	No hit	unanchored	419	140	+	Regular	$MIKC^C$	Dispersed	AT4G09960.1	238	1.00E-63
MdMADS139	MdMADS139	MDP0000278738	Contig_33460	unanchored	771	256	+	Regular	$MIKC^C$	Segmental	AT5G23260.3	167	5.00E-42
MdMADS140	MdMADS140	MDP0000314422	No hit	unanchored	1833	610	-	Non-regular	Mα	Proximal	AT2G24840.1	117	2.00E-26
MdMADS141	MdMADS141	MDP0000434703	No hit	unanchored	684	228	-	Regular	Mα	Tandem	AT5G60440.1	227	6.00E-60
MdMADS142	MdMADS142	MDP0000753870	No hit	unanchored	1086	362	-	Regular	Mβ	Dispersed	AT5G55690.1	98	1.00E-20

Supplementary Table S2. Comparison of MdMADS identified in present study with that of MdMADS identified by Tian et al. (2015).

Present study	Tian et al., 2015		Previously identified MdMADS acession no.
**Present nomenclature**	**Dong et al., nomenclature**	**MDP ID**	
MdMADS001	MdMADS133	MDP0000152718	
MdMADS002			
MdMADS003	MdMADS139	MDP0000146415	
MdMADS004	MdMADS132	MDP0000133671	
MdMADS005	MdMADS80	MDP0000919323	
MdMADS006	MdMADS73	MDP0000144597	
MdMADS007	MdMADS75	MDP0000462145	
MdMADS008	MdMADS21	MDP0000249592	HM122605
MdMADS009	MdMADS65	MDP0000449389	
MdMADS010	MdMADS63	MDP0000200272	
MdMADS011	MdMADS101	MDP0000696331	
MdMADS012	MdMADS13	MDP0000315938	AJ251116
MdMADS013	MdMADS39	MDP0000184294	
MdMADS014	MdMADS26	MDP0000314765	
MdMADS015	MdMADS11	MDP0000149676	AJ000763
MdMADS016	MdMADS88	MDP0000237091	
MdMADS017	MdMADS144	MDP0000162019	
MdMADS018		MDP0000123878	
MdMADS019		MDP0000726295	
MdMADS020	MdMADS121	MDP0000309181	
MdMADS021	MdMADS32	MDP0000156783	
MdMADS022	MdMADS31	MDP0000136650	
MdMADS023	MdMADS92	MDP0000155389	
MdMADS024		MDP0000209705	
MdMADS025	MdMADS134	MDP0000212925	
MdMADS026	MdMADS131	MDP0000129298	
MdMADS027	MdMADS22	MDP0000250080	HM122606
MdMADS028	MdMADS83	MDP0000120467	
MdMADS029	MdMADS102	MDP0000696333	
MdMADS030	MdMADS106	MDP0000183226	
MdMADS031	MdMADS105	MDP0000181583	
MdMADS032	MdMADS112	MDP0000179718	
MdMADS033	MdMADS107	MDP0000234073	
MdMADS034	MdMADS40	MDP0000224779	
MdMADS035	MdMADS86	MDP0000233925	
MdMADS036	MdMADS87	MDP0000233926	
MdMADS037	MdMADS90	MDP0000278770	
MdMADS038	MdMADS53	MDP0000228942	
MdMADS039	MdMADS54	MDP0000475549	
MdMADS040	MdMADS137	MDP0000451827	
MdMADS041	MdMADS125	MDP0000299637	
MdMADS042	MdMADS135	MDP0000236438	
MdMADS043	MdMADS124	MDP0000664841	
MdMADS044	MdMADS38	MDP0000133037	
MdMADS045	MdMADS71	MDP0000312382	
MdMADS046	MdMADS48	MDP0000132226	
MdMADS047		MDP0000670559	
MdMADS048	MdMADS79	MDP0000193129	
MdMADS049	MdMADS6	MDP0000574222	AJ000760
MdMADS050	MdMADS36	MDP0000218020	
MdMADS051	MdMADS33	MDP0000254052	
MdMADS052		MDP0000216605	

MdMADS053	MdMADS110	MDP0000127771	
MdMADS054	MdMADS68	MDP0000148857	
MdMADS055	MdMADS59	MDP0000783966	
MdMADS056	MdMADS64	MDP0000060753	
MdMADS057	MdMADS23	MDP0000220168	HM122607
MdMADS058	MdMADS76	MDP0000152821	
MdMADS059	MdMADS77	MDP0000243965	
MdMADS060	MdMADS24	MDP0000286643	
MdMADS061	MdMADS43	MDP0000259294	
MdMADS062	MdMADS44	MDP0000259296	
MdMADS063	MdMADS20	MDP0000527190	HM122604
MdMADS064	MdMADS47	MDP0000378412	
MdMADS065	MdMADS128	MDP0000925348	
MdMADS066	MdMADS72	MDP0000315764	
MdMADS067	MdMADS60	MDP0000193953	
MdMADS068	MdMADS62	MDP0000192806	
MdMADS069	MdMADS120	MDP0000296123	
MdMADS070	MdMADS9	MDP0000289858	AJ001682
MdMADS071	MdMADS30	MDP0000605482	
MdMADS072	MdMADS37	MDP0000132738	
MdMADS073	MdMADS51	MDP0000143531	
MdMADS074	MdMADS41	MDP0000223851	
MdMADS075	MdMADS14	MDP0000324259	AJ251117
MdMADS076	MdMADS95	MDP0000611204	
MdMADS077	MdMADS91	MDP0000291070	
MdMADS078	MdMADS34	MDP0000518032	
MdMADS079	MdMADS136	MDP0000254632	
MdMADS080		MDP0000123808	
MdMADS081	MdMADS17	MDP0000270062	HM122600
MdMADS082	MdMADS67	MDP0000248130	
MdMADS083	MdMADS69	MDP0000205637	
MdMADS084	MdMADS89	MDP0000274606	
MdMADS085	MdMADS15	MDP0000324166	AJ251118
MdMADS086	MdMADS61	MDP0000316609	
MdMADS087	MdMADS46	MDP0000311107	
MdMADS089	MdMADS96	MDP0000314058	
MdMADS088	MdMADS50	MDP0000233948	
MdMADS090	MdMADS49	MDP0000233843	
MdMADS091	MdMADS99	MDP0000523644	
MdMADS092	MdMADS103	MDP0000713024	
MdMADS093	MdMADS85	MDP0000232338	
MdMADS094	MdMADS126	MDP0000307605	
MdMADS095	MdMADS122	MDP0000834768	
MdMADS096	MdMADS129	MDP0000599449	
MdMADS097	MdMADS35	MDP0000269921	
MdMADS098	MdMADS4	MDP0000326906	U78950
MdMADS099	MdMADS78	MDP0000146231	
MdMADS100	MdMADS29	MDP0000370413	
MdMADS101	MdMADS55	MDP0000285581	
MdMADS102	MdMADS142	MDP0000229755	
MdMADS103	MdMADS84	MDP0000221590	
MdMADS104	MdMADS138	MDP0000296791	
MdMADS105	MdMADS97	MDP0000287530	
MdMADS106	MdMADS145	MDP0000289392	
MdMADS107	MdMADS82	MDP0000773068	
MdMADS108	MdMADS57	MDP0000135652	
MdMADS109	MdMADS58	MDP0000144516	
MdMADS110	MdMADS143	MDP0000322264	
MdMADS111	MdMADS70	MDP0000300752	
MdMADS112	MdMADS3/7	MDP0000326390	U78949/HM122610

MdMADS113	MdMADS2	MDP0000289836	U78948
MdMADS114	MdMADS130	MDP0000846004	
MdMADS115	MdMADS56	MDP0000234911	
MdMADS116	MdMADS116	MDP0000134211	
MdMADS117	MdMADS118	MDP0000186886	
MdMADS118	MdMADS119	MDP0000192342	
MdMADS119	MdMADS141	MDP0000303501	
MdMADS120	MdMADS5	MDP0000013331	AJ000759
MdMADS121	MdMADS27	MDP0000280009	
MdMADS122	MdMADS81	MDP0000147667	
MdMADS123	MdMADS28	MDP0000220008	
MdMADS124	MdMADS42	MDP0000255146	
MdMADS125	MdMADS52	MDP0000322567	
MdMADS126	MdMADS45	MDP0000277582	
MdMADS127	MdMADS1/8	MDP0000366022	U78947/AJ001681
MdMADS128	MdMADS25	MDP0000242009	
MdMADS129	MdMADS104	MDP0000315688	
MdMADS130	MdMADS114	MDP0000128016	
MdMADS131	MdMADS111	MDP0000138447	
MdMADS132	MdMADS93	MDP0000159062	
MdMADS133	MdMADS94	MDP0000168914	
MdMADS134	MdMADS117	MDP0000185853	
MdMADS135	MdMADS108	MDP0000186558	
MdMADS136	MdMADS109	MDP0000218227	
MdMADS137	MdMADS140	MDP0000251651	
MdMADS138	MdMADS10	MDP0000268317	AJ000762
MdMADS139	MdMADS113	MDP0000278738	
MdMADS140	MdMADS100	MDP0000314422	
MdMADS141	MdMADS98	MDP0000434703	
MdMADS142	MdMADS123	MDP0000753870	
	MdMADS19	MDP0000120269	HM122602
	MdMADS146	MDP0000167796	
	MdMADS115	MDP0000190058	
	MdMADS127	MDP0000228437	
	MdMADS16	MDP0000233948	HM122599
	MdMADS66	MDP0000270062	
	MdMADS12	MDP0000278897	AJ320187
	MdMADS74	MDP0000904420	
	MdMADS18	MDP0000936232	HM122601

Reported in present study only
Reported by Tian et al. only
Reported by Tian et al. only, however contains only K domain
Same MdMADSs reported twice by Tian et al.

Supplementary Table S3. The sample abbreviation and their collection intervals.

**Developmental stages/Tissues**	**Sample code**	**Collection intervals**
**Apical bud**	AP1	0 day
	AP2	38 days
	AP3	70 days
**Spur bud**	SP1	0 day
	SP2	38 days
	SP3	70 days
	SP4	104 days
	SP5	126 days
**Bud break**	GT (Green tip)	0 day
**Flower**	TC (Tight cluster)	10 days
	IP (Initial pink)	15 days
	FP (Full pink)	26 days
	FF (Bloom)	30 days
	PF (Petal fall)	39 days
**Fruit**	FS1	13 DAP
	FS2	28 DAP
	FS3	44 DAP
	FS4	83 DAP
	FS5	102 DAP
	FS6	132 DAP
	FS7	161 DAP

Supplementary Table S4. List of low temperature responsive elements in 1.5Kb upstream promoter regions of MdDAMs.

DAM genes	Element name	Up-stream transcription start Site	Strand	Motif sequence	PLACE motif ID
MdMADS024	LTRECOREATCOR15	125	(+)	CCGAC	S000153
MdMADS061	LTRECOREATCOR15	1167	(+)	CCGAC	S000153
MdMADS063	LTRECOREATCOR15	278	(+)	CCGAC	S000153
	LTRECOREATCOR15	308	(+)	CCGAC	S000153
MdMADS088	No LTRE element found				
MdMADS124	No LTRE element found				
MdMADS125	CBFHV	745	(+)	RYCGAC	S000497
	CBFHV	833	(+)	RYCGAC	S000497
	CBFHV	540	(+)	RYCGAC	S000497
	LTREATLTI78	833	(+)	ACCGACA	S0157
	LTRECOREATCOR15	266	(+)	CCGAC	S000153
	LTRECOREATCOR15	541	(+)	CCGAC	S000153
	LTRECOREATCOR15	834	(+)	CCGAC	S000153

Supplementary Table S5. List of DNA binding site sequence for MdMADS proteins identified in apple.

Repoductive phase SVP binding motifs	Vegetative phase SVP binding motif
ccaaaaaagg	ccaaaaaagg
ccaaaaatgg	ccaaaaatgg
ccaaaattgg	ccaaaattgg
ccaaattagg	ccaaatatgg
ccaaatttgg	ccaaatttgg
ccaatattgg	ccaatattgg
ccaattttgg	ccaattatgg
ccataaatgg	ccaatttagg
ccattaaagg	ccataattgg
ccattaatgg	ccatattagg
ccatttttag	ccatatttgg
ccatttttgg	ccattattgg
cctaaaaagg	ccattttagg
cctaattagg	cctaaaaagg
cctataatgg	cctaaattgg
cctattaagg	cctaatttgg
ccttaaaagg	cctataatgg
ccttattagg	cctattaagg
ccttatttgg	ccttaaaagg
ccttttatgg	ccttaatagg
cctttttagg	ccttaattgg
ccttttttgg	cctttaatgg
	ccttttttgg

Supplementary Table S6. List of hub proteins and their interacting partners in Arabidopsis identified through protein-protein interaction network and their orthologs in apple.

At hub gene	At hub gene interactor	Putative apple hub gene (At ortholog)	Putative apple hub gene interactor (At ortholog)
**AT1G66410**		**MDP0000344216**	
	AT5G49450		MDP0000335904
	AT1G76650		MDP0000301599
	AT1G09840		MDP0000168962
	AT1G02920		MDP0000212253
	AT1G69270		MDP0000224837
	AT4G02640		MDP0000945064
	AT4G23650		MDP0000261109
	AT5G60910		MDP0000173549
	AT1G48090		MDP0000242105
	AT4G04570		MDP0000306767
	AT1G32250		MDP0000499214
	AT4G18630		MDP0000186456
	AT4G18950		MDP0000892891
	AT5G17470		MDP0000892891
	AT3G61650		MDP0000743939
**AT5G23290**		**MDP0000218129**	
	AT5G19820		MDP0000770853
	AT3G12810		MDP0000129826
	AT1G54960		MDP0000861495
	AT5G55310		MDP0000893502
	AT4G29810		MDP0000251096
	AT2G18990		MDP0000248767
	AT1G14690		MDP0000123927
	AT4G14960		MDP0000829998
	AT2G36200		MDP0000158090
	AT3G61650		MDP0000743939
	AT4G39050		MDP0000162639
	AT3G47690		MDP0000317112
**AT5G19820**		**MDP0000770853**	
	AT3G16950		MDP0000310711
	AT4G14960		MDP0000829998
	AT1G07660		MDP0000923289
	AT3G05960		MDP0000529326
	AT3G51260		MDP0000326966
	AT5G23290		MDP0000218129
	AT3G19980		MDP0000172963
	AT1G70490		MDP0000788110
	AT5G02530		MDP0000142206
	AT4G34910		MDP0000308104
	AT3G01280		MDP0000301544
**AT3G19980**		**MDP0000172963**	
	AT3G16950		MDP0000310711
	AT3G05960		MDP0000529326
	AT5G19820		MDP0000770853
	AT1G09840		MDP0000168962
	AT4G21800		MDP0000201702
	AT4G05020		MDP0000120459
	AT5G55260		MDP0000266741
	AT3G01280		MDP0000301544
**AT4G14960**		**MDP0000829998**	
	AT5G23670		MDP0000251269
	AT5G02880		MDP0000699611
	AT5G59840		MDP0000315756
	AT5G19820		MDP0000770853
	AT5G63860		MDP0000794968
	AT5G23290		MDP0000218129
	AT3G61650		MDP0000743939
	AT5G07760		MDP0000311544

Supplementary Table S7. Over-represented biological process and molecular function gene ontology terms associated with reproductive phase network proteins.

Biological process			Molecular function
	**Post-embryonic development (p value; 0.00171)**		**Protein kinase activity (p value; 0.0112)**
**At ID**	**Go terms**	**At ID**	**Go terms**
AT3G19980	GO:0005737 cytoplasm<br>GO:0000159 protein phosphatase type 2A complex<br>GO:0005634 nucleus<br>GO:0009910 negative regulation of flower development<br>GO:0004722 protein serine/threonine phosphatase activity<br>GO:0004674 protein serine/threonine kinase activity<br>GO:0009793 embryonic development ending in seed dormancy<br>GO:0005515 protein binding	AT3G19980	GO:0005737 cytoplasm<br>GO:0000159 protein phosphatase type 2A complex<br>GO:0005634 nucleus<br>GO:0009910 negative regulation of flower development<br>GO:0004722 protein serine/threonine phosphatase activity<br>GO:0004674 protein serine/threonine kinase activity<br>GO:0009793 embryonic development ending in seed dormancy<br>GO:0005515 protein binding
AT5G60910	GO:0003700 transcription factor activity<br>GO:0009911 positive regulation of flower development<br>GO:0010154 fruit development	AT1G69270	GO:0004702 receptor signaling protein serine/threonine kinase activity<br>GO:0009738 abscisic acid mediated signaling pathway<br>GO:0005515 protein binding<br>GO:0005886 plasma membrane
AT3G12810	GO:0048451 petal formation<br>GO:0003677 DNA binding<br>GO:0004386 helicase activity<br>GO:0042742 defense response to bacterium<br>GO:0016514 SWI/SNF complex<br>GO:0005524 ATP binding	AT4G29810	GO:0005737 cytoplasm<br>GO:0009814 defense response, incompatible interaction<br>GO:0009631 cold acclimation<br>GO:0000165 MAPKKK cascade<br>GO:0009651 response to salt stress<br>GO:0004708 MAP kinase kinase activity
AT3G61650	GO:0009553 embryo sac development<br>GO:0000914 phragmoplast assembly<br>GO:0048768 root hair cell tip growth<br>GO:0005739 mitochondrion<br>GO:0005198 structural molecule activity<br>GO:0048366 leaf development<br>GO:0005874 microtubule<br>GO:0010103 stomatal complex morphogenesis<br>GO:0051641 cellular localization	AT4G18950	GO:0005737 cytoplasm<br>GO:0009966 regulation of signal transduction<br>GO:0005634 nucleus<br>GO:0004712 protein serine/threonine/tyrosine kinase activity<br>GO:0006468 protein amino acid phosphorylation
AT5G49450	GO:0003700 transcription factor activity<br>GO:0006355 regulation of transcription, DNA-dependent<br>GO:0046982 protein heterodimerization activity<br>GO:0009901 anther dehiscence	AT1G54960	GO:0000910 cytokinesis<br>GO:0004709 MAP kinase kinase kinase activity
AT5G23670	GO:0005783 endoplasmic reticulum<br>GO:0009555 pollen development<br>GO:0016020 membrane<br>GO:0004758 serine C-palmitoyltransferase activity<br>GO:0005515 protein binding<br>GO:0046512 sphingosine biosynthetic process<br>GO:0009640 photomorphogenesis	AT1G09840	GO:0004672 protein kinase activity
AT5G19820	GO:0016829 lyase activity<br>GO:0030089 phycobilisome<br>GO:0009793 embryonic development ending in seed dormancy	AT4G23650	GO:0006468 protein amino acid phosphorylation<br>GO:0004683 calmodulin-dependent protein kinase activity<br>GO:0009738 abscisic acid mediated signaling pathway<br>GO:0010119 regulation of stomatal movement<br>GO:0010359 regulation of anion channel activity<br>GO:0005886 plasma membrane

Supplementary Table S8. List of primer sequence of randomly selected 10 MdMADS genes.

Sr. No	Gene name/clade	MdMADS ID	Primer sequence
1	DAM	*MdMADS125*	Fwd 5'-GAGTGAGGTTATGGCACTTGAGAA-3'
			Rev 5'-GATGGCCTCTGGTCCAATATCTC-3'
2	DAM	*MdMADS024*	Fwd 5'-CCAGCTCTAGTATGAAGGAAA-3'
			Rev 5'-TCTCCACTAGCTGTAACTCAA-3'
3	TM8	*MdMADS111*	Fwd 5'-CGACATAACTAGGACCATTTC-3'
			Rev 5'-CCTCCAATACTCCATTGTTCT-3'
4	AP3/PI	*MdMADS044*	Fwd 5'-TGGAGGAAAGAAGAGGAAACATG-3'
			Rev 5'-TCTCCCTCATTGTCCACATAACAA-3'
5	SEP	*MdMADS123*	Fwd 5'-TGTCGCTGAAGCAGATCAGATC-3'
			Rev 5'-CTTCGTTTAGCACGTGTTCATTTC-3'
6	MIKCC-II	*MdMADS126*	Fwd 5'-GGCAACAACATTTAATCCATCGA-3'
			Rev 5'-GAACTCCCACCCGTCATAACC-3'
7	FLC-like	*MdMADS135*	Fwd 5'-GTCTCAGATTGGGTGCATTGT-3'
			Rev 5'-GGCTAACTTAAATCTGGTGCAACA-3'
8	SOC1	*MdMADS014*	Fwd 5'-GGGAACCATAGAGCGTTATCAGAA-3'
			Rev 5'-TTCAGATGCTGCATATTTTGTTCA-3'
9	AP1	*MdMADS050*	Fwd 5'-CTTCTCTACCAAAGGCAAGCTTTT-3'
			Rev 5'-CACCAGCACTATACCCAGTCTGTCT-3'
10	AGL15	*MdMADS051*	Fwd 5'-AGCTTCGATGTTTGTTTCCACAA-3'
			Rev 5'-GGTTTACAAGGGATTTGGTTTTCTC-3'
